# cfMethylPre: deep transfer learning enhances cancer detection based on circulating cell-free DNA methylation profiling

**DOI:** 10.1093/bib/bbaf303

**Published:** 2025-06-29

**Authors:** Xuchao Zhang, Jing Chen, Yongtian Wang, Xiaofeng Wang, Jialu Hu, Jiajie Peng, Xuequn Shang, Yanpu Wang, Tao Wang

**Affiliations:** School of Computer Science, Northwestern Polytechnical University, 1 Dongxiang Rd., Xi’an 710072, Shaanxi, China; Key Laboratory of Big Data Storage and Management, Ministry of Industry and Information Technology, Northwestern Polytechnical University, 1 Dongxiang Rd., Xi’an 710072, Shaanxi, China; School of Computer Science and Engineering, Xi’an University of Technology, No. 5 South Jinhua Road, Xi’an 710018, Shaanxi, China; School of Computer Science, Northwestern Polytechnical University, 1 Dongxiang Rd., Xi’an 710072, Shaanxi, China; Key Laboratory of Big Data Storage and Management, Ministry of Industry and Information Technology, Northwestern Polytechnical University, 1 Dongxiang Rd., Xi’an 710072, Shaanxi, China; General Surgery Department, The Afliated Hospital of Northwest University, Xi’an No 3 Hospital, No.5 South Jinhua Rd., Xi’an 710048, Shaanxi, China; School of Computer Science, Northwestern Polytechnical University, 1 Dongxiang Rd., Xi’an 710072, Shaanxi, China; Key Laboratory of Big Data Storage and Management, Ministry of Industry and Information Technology, Northwestern Polytechnical University, 1 Dongxiang Rd., Xi’an 710072, Shaanxi, China; School of Computer Science, Northwestern Polytechnical University, 1 Dongxiang Rd., Xi’an 710072, Shaanxi, China; Key Laboratory of Big Data Storage and Management, Ministry of Industry and Information Technology, Northwestern Polytechnical University, 1 Dongxiang Rd., Xi’an 710072, Shaanxi, China; School of Computer Science, Northwestern Polytechnical University, 1 Dongxiang Rd., Xi’an 710072, Shaanxi, China; Key Laboratory of Big Data Storage and Management, Ministry of Industry and Information Technology, Northwestern Polytechnical University, 1 Dongxiang Rd., Xi’an 710072, Shaanxi, China; Cancer Institute (Key Laboratory of Cancer Prevention and Intervention, China National Ministry of Education), The Second Affiliated Hospital, Zhejiang University School of Medicine, Hangzhou 310000, Zhejiang, China; Cancer Center of Zhejiang University, Hangzhou 310000, Zhejiang, China; School of Computer Science, Northwestern Polytechnical University, 1 Dongxiang Rd., Xi’an 710072, Shaanxi, China; Key Laboratory of Big Data Storage and Management, Ministry of Industry and Information Technology, Northwestern Polytechnical University, 1 Dongxiang Rd., Xi’an 710072, Shaanxi, China

**Keywords:** cell-free DNA methylation, deep learning, transfer learning, large language model

## Abstract

Cancer remains a significant global health burden, underscoring the need for innovative diagnostic tools to enable early detection and improve patient outcomes. While circulating cell-free DNA (cfDNA) methylation has emerged as a promising biomarker for noninvasive cancer diagnostics, existing methods often face limitations in handling the high-dimensionality of methylation data, small sample sizes, and a lack of biological interpretability. To address these challenges, we propose cfMethylPre, a novel deep transfer learning framework tailored for cancer detection using cfDNA methylation data. cfMethylPre leverages large language model pretrained embeddings from DNA sequence information and integrates them with methylation profiles to enhance feature representation. The deep transfer learning process involves pretraining on bulk DNA methylation data encompassing 2801 samples across 82 cancer types and normal controls, followed by fine-tuning with cfDNA methylation data. This approach ensures robust adaptation to cfDNA’s unique characteristics while improving predictive accuracy. Our model achieved superior predictive accuracy compared with state-of-the-art methods, with a weighted Matthews Correlation Coefficient of 0.926 and a weighted F1-score of 0.942. Through model interpretation and biological experimental validation, we identified three novel breast cancer genes—*PCDHA10*, *PRICKLE2*, and *PRTG*—demonstrating their inhibitory effects on cell proliferation and migration in breast cancer cell lines. These findings establish cfMethylPre as a powerful and interpretable tool for cancer diagnostics and biological discovery, paving the way for its application in precision oncology.

## Introduction

Cancer continues to be a leading cause of morbidity and mortality globally, highlighting the vital need for early detection to enhance patient outcomes [1, 2]. Although traditional diagnostic approaches, including imaging and tissue biopsies, are effective, they present challenges related to sensitivity, specificity, timeliness, and invasiveness [3]. Consequently, there is a significant impetus to innovate noninvasive diagnostic techniques that can detect cancer at its most incipient stage.

In recent years, cell-free DNA (cfDNA) holds promise as a potential biomarker for detecting cancer [4]. cfDNA consists of fragmented DNA released into the bloodstream by normal and cancerous cells, offering a unique opportunity for noninvasive cancer diagnostics, often referred to as “liquid biopsies” [5]. Unlike traditional tissue biopsies, liquid biopsies are minimally invasive, can be repeated, and provide a comprehensive picture of the entire tumor genome, including its heterogeneity [6]. DNA methylation, an epigenetic modification involving the addition of a methyl group to cytosine residues in CpG dinucleotides, plays a crucial role in regulating gene expression [7, 8]. Aberrant methylation patterns are a hallmark of cancer, with tumor suppressor genes often experiencing hypermethylation and oncogenes undergoing hypomethylation [9]. Analyzing these methylation patterns in cfDNA presents a powerful biomarker for noninvasive cancer detection and classification, with the potential to uncover underlying mechanisms of tumor development and progression. Another key advantage of using cfDNA methylation as a cancer biomarker is the stability of DNA methylation when compared with other biomarkers, such as genetic mutations [10]. For instance, the methylation of the 14-3-3 sigma promoter, which is present in 96% of breast cancer cases, clearly distinguishes it from the absence of methylation in healthy breast epithelium [11]. The robustness is also seen in high-grade serous ovarian cancer, where promoters like *HOXA9* and *EN1* consistently exhibit hypermethylation in the majority of cases [12].

Extensive researches highlight cfDNA methylation as a promising biomarker for cancer diagnostics, with advancements in both statistical and machine learning methods [13]. For example, Liang *et al*. [14] identified differentially methylated regions in ovarian cancer cfDNA, while Škara *et al*. [15] analyzed methylation in the Caveolin-1 (CAV1) gene for prostate cancer detection. Machine learning has further enhanced these efforts: Zhao *et al*. [16] and Xu *et al*. [17] applied models like XGBoost and random forest to cfDNA methylation data for colorectal and multi-cancer classification, respectively. Advanced frameworks, such as Kim *et al*.’s [18] deep learning approach using EM-seq for lung cancer and Heeke *et al*.’s [19] methylation-based classifier for small cell lung cancer, have demonstrated improved sensitivity and specificity [20–22]. Moreover, tools like MethylNet and ensemble methods integrating algorithms such as support vector machines (SVMs) and random forests continue to refine diagnostic accuracy [23, 24]. These innovations underscore the transformative potential of cfDNA methylation data, positioning it as a cornerstone for future cancer diagnostics [25].

Despite these advancements, several challenges persist. Many studies are constrained by small sample sizes, which limit the robustness and generalizability of their findings. Traditional machine learning models also struggle to effectively capture the complex patterns inherent in high-dimensional methylation data, leading to suboptimal predictive performance. Moreover, a critical limitation of current approaches is their lack of interpretability; most models fail to account for the functional genes associated with methylation sites, making it difficult to connect predictions with biological relevance. Although some methods attempt to infer gene associations, biological validation is often insufficient, further impeding clinical translation. These limitations not only restrict the practical application of such models in diagnostics but also diminish their potential to enhance our understanding of the molecular mechanisms underlying cancer.

In this study, we present cfMethylPre, a novel deep transfer learning framework designed to predict multiple cancers using cfDNA methylation data. Our transfer learning strategy involves pretraining the model on bulk DNA methylation data from an extensive integrated cohort comprising 2801 samples across 82 cancer types and nine types of normal controls. This pretrained model is then fine-tuned using cfDNA methylation data, enabling it to adapt effectively to the unique characteristics of cfDNA. Unlike traditional approaches, our deep learning framework incorporates both DNA methylation profiles and DNA sequence information, with the latter derived from a pretrained large language model, enhancing the model’s ability to capture complex genomic patterns [26–28]. Additionally, cfMethylPre provides interpretability, allowing us to identify genes with high susceptibility to cancer. Using this capability, we detected and validated three genes—*PCDHA10*, *PRICKLE2*, and *PRTG*—demonstrating their inhibiting effects on cancer cell growth and migration in breast cancer cell lines. These advancements establish cfMethylPre as a powerful and interpretable tool for cancer diagnostics and biological discovery.

## Materials and methods

### Architecture of cfMethylPre

**Figure 1:**
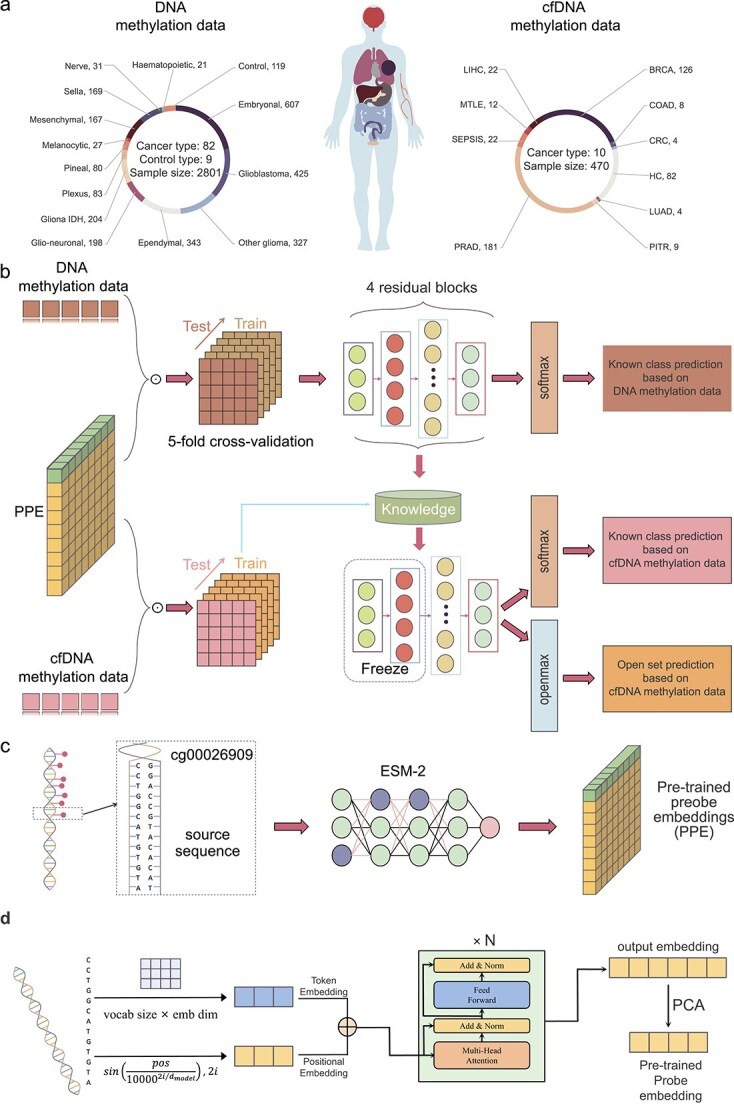
The framework of cfMethylPre. (a) Overview of training data, comprising 2801 DNA methylation samples and 470 cfDNA methylation samples. (b) The training workflow involves reconstructing DNA and cfDNA methylation data using PPE, pretraining on DNA methylation via five-fold crossover, fine-tuning for cfDNA methylation, and outputting both traditional and unknown class predictions. (c) Encoding process using the pretrained model for PPE generation (d) Details of generating PPE using ESM-2.

As shown in Fig. 1, cfMethylPre adopts a transfer learning framework, initially training the model using bulk-DNA methylation data as the source domain and subsequently fine-tuning it with cfDNA methylation data as the target domain. The model is trained on enhanced methylation features, which integrate methylation profiles with local DNA sequence embeddings. In the following context, we will introduce three key modules in the cfMethylPre framework.

#### Encoding DNA sequence information with large language model

To incorporate sequence-based context into our analysis, we first mapped each probe site to its corresponding nucleotide sequence. We then employed ESM-2, a pretrained large-scale biological language model originally designed for protein encoding, to encode the DNA sequence information surrounding each methylation probe site into a latent vector representation [29]. For each probe, we retrieved a 50-base pair (bp) DNA sequence, comprising 25 bp upstream and 25 bp downstream of the probe site.

The ESM-2 model comprises 30 layers and 150 million parameters, specifically designed to extract insights from sequences. It functions by predicting actual residues in masked segments of a sequence based on the surrounding visible ones, thereby learning meaningful representations of data. This capability enables the model to capture both local and global patterns within sequences. As depicted in Fig. 1d, the sequence encoding process for individual CpG sites begins by transforming the raw sequence into token and positional embeddings using a predefined vocabulary. These embeddings are then processed through ESM-2’s multi-layer transformer module to generate initial output embeddings.

Using the pretrained model, each DNA sequence was encoded into a vector with 640 dimensions. To manage high dimensionality and enhance computational efficiency, principal component analysis (PCA) was applied to reduce the feature space to 128 dimensions, retaining the most informative components of the encoded data. The transformation can be expressed mathematically as follows:


(1) \begin{align*}& v_{i} = \text{ESM-2}(s_{i}) = e_{1}, e_{2}, \ldots, e_{640}\end{align*}

Here, $s_{i}$ denotes the DNA sequence of the methylation probe, $v_{i}$ is the encoded vector produced by the pretrained model, and $e_{i}$ denotes the 640-dimensional output obtained after encoding the sequence using ESM-2. Subsequently, PCA is employed to reduce the dimensionality of $v_{i}$ to 128 dimensions for further computations.

This process yielded a matrix of pretrained probe embeddings (PPE), incorporating sequence information, with dimensions $6585 \times 128$. Each methylation beta value at a given locus was further enriched by multiplying it by the elements of its corresponding encoding vector. Through this operation, we fused the methylation feature and the local DNA sequence information, which will be used for model training in the following.

#### Transfer learning for cfDNA methylation adaptation

The ResNet structure serves as the backbone of cfMethylPre, utilizing four blocks comprising 3, 4, 23, and 3 Bottleneck units, respectively. As part of the transfer learning strategy, after training in the source domain, the parameters in the first two blocks are fixed, while the parameters in the remaining two blocks are fine-tuned using the target domain data.

In detail, each block’s first Bottleneck unit consists of both a main path and a downsampling path. The main path includes three convolutional layers: a 1$\times $1 convolution reducing channels from 1024 to 512, a 3$\times $3 convolution with stride 2 maintaining 512 channels, and a final 1$\times $1 convolution expanding channels from 512 to 2048. Each convolution is followed by a BatchNorm2d layer and a ReLU activation function. The downsampling path features a 1$\times $1 convolution with a stride 2 that adjusts the input channels from 1024 to 2048, followed by a BatchNorm2d layer. The outputs of the main and downsampling paths are then added together. Subsequent Bottleneck units within each block maintain a similar structure, with the second convolutional layer using a stride of 1 and omitting the downsampling path. A key component of the architecture is the residual block with skip connections, which facilitates smooth propagation of the gradient throughout the network, significantly improving the efficiency and stability of the model training. The training strategy—including the optimizer, learning rate, and loss function—was kept consistent between pretraining on DNA methylation data and fine-tuning on cfDNA methylation data to ensure stable and comparable model performance.

#### Prediction of unknown cancer types

To enhance cfMethylPre’s ability to predict cfDNA methylation patterns in scenarios with unlabeled data, an unknown class prediction module is introduced, leveraging the OpenMax open set identification algorithm, which extends softmax by incorporating the likelihood of unrecognized categories [30]. To improve categorical accuracy, cfMethylPre utilizes distance distributions based on extreme value theory and the Weibull distribution for confidence assessment [31]. The model first applies softmax to raw data, generating activation vectors $AV_{i} = {AV_{1}, AV_{2}, \ldots , AV_{m}}$, then calculates distance distributions $D_{i} = {D_{1}, D_{2}, \ldots , D_{m}}$, representing the dissimilarities between the derived and average activation vectors. These distances are used to compute category-specific confidence levels.


(2) \begin{align*}& w_{j}=1-Score_{w}(D_{x_{j}})\end{align*}

Adjustments to the softmax output are made accordingly:


(3) \begin{align*} & Score_{j^{\prime}}=Score_{j^{\prime}}\times w_{j} \end{align*}

(4) \begin{align*} & Score_{unknown}=\sum_{i=1}^{j}Score_{i}\times \left ( 1-w_{i} \right ) \end{align*}

This iterative refinement produces an updated score vector $Score_{1^{\prime}}$, $Score_{2^{\prime}}$,..., $Score_{K^{\prime}}$, $Score_{unknown}$, where if $Score_{unknown}$ is the highest, the sample is classified as the unknown category. The full ResNet architecture and the workflow of processing a single sample are provided in Supplementary Figure 1.

### Model training and evaluation

To rigorously evaluate model performance, cfMethylPre utilizes a five-fold cross-validation strategy. The dataset is partitioned into five equal subsets, with four used for training and one reserved for testing in each iteration. This process is rotated across five cycles to ensure a comprehensive evaluation. During each cycle, fine-tuning is applied to the final two layers of the model, enabling domain-specific optimization for the target dataset. The training process is mathematically defined as


(5) \begin{align*} & f\left ( x \right ) =W_{L}\sigma \left ( H_{L-1}+F_{L-1}\left ( H_{L-1}\right ) \right ) +b_{L} \end{align*}

(6) \begin{align*} & F_{l}\left ( H_{l} \right )=W_{L,2}\sigma \left ( W_{l,1}+b_{l,1} \right ) +b_{l,2} \end{align*}

Here, $x$ denotes the input features, $f\left ( x \right )$ represents the model output, $W_{L}$ and $b_{L}$ are the weights and biases of the last layer, and $\sigma $ is the activation function. The terms $H_{L-1}$ and $F_{L-1}$ represent the outputs and functions of preceding layers. $ W_{l,1} $ and $ W_{l,2} $ represent the weight matrices for the first and second transformations within the function $ F_{l} $ at layer $ l $, respectively, while $ b_{l,1} $ and $ b_{l,2} $ denote the corresponding bias vectors for these transformations. The output from layer $ l $ is denoted by $ H_{l} $. The function $ F_{l}(H_{l}) $ performs a two-step transformation on $ H_{l} $ by applying these weight matrices and biases, followed by the activation function $ \sigma $.

The model’s performance is further optimized using a customized loss function tailored for multi-class classification of methylation data:


(7) \begin{align*}& L=-\frac{1}{n}\sum_{i=1}^{n}\sum_{c=1}^{C} y_{ic} \log \frac{\exp(f_{c}(x_{i}))}{\sum_{j=1}^{C} \exp(f_{j}(x_{i}))}\end{align*}

In this equation, $n$ is the total number of samples, $C$ represents the number of categories, and $y_{ic}$ is a binary indicator (0 or 1) signaling whether class label $c$ is correct for sample $i$. The terms $f_{c}(x_{i})$ and $f_{j}(x_{i})$ denote the predicted probabilities for each category.

The model was trained with a learning rate of 1e-5, a batch size of 16, and optimized using the Adam optimizer; early stopping was triggered if validation loss showed no improvement for 10 consecutive epochs.

### Data collection and preprocessing

The cfDNA methylation data and DNA methylation data used in this study were obtained from the Gene Expression Omnibus (GEO, https://www.ncbi.nlm.nih.gov/geo) and the Cell-Free Epigenome Atlas (CFEA, http://www.bio-data.cn/CFEA). After data acquisition, the samples underwent a standardized preprocessing protocol to compute methylation beta values, which reflect methylation levels at specific CpG sites throughout the genome [32]. The methylation beta value, a critical metric in our analysis, is calculated using the formula:


(8) \begin{align*}& \beta = \frac{M}{M + U + \textrm{offset}}\end{align*}

Here, $M$ represents the methylated signal intensity, while $U$ represents the unmethylated signal intensity. An offset was introduced to prevent division by zero, thereby ensuring stability in the calculation of methylation levels. Quality control was conducted using $P$-values as a filtering criterion. Beta values with corresponding $P$-values exceeding.01 were considered below the acceptable intensity threshold and were set to “NA.”

In the subsequent genome-wide DNA methylation data analysis, we first identified CpG sites with significant variability. To enhance computational efficiency and streamline the training process, the top 10 000 sites were selected using the Random Forest algorithm [33]. This selection was based solely on DNA methylation data, ensuring no label leakage from the cfDNA methylation data in the target domain.

To ensure data integrity and analytical accuracy, we implemented a rigorous preprocessing pipeline where CpG sites with missing methylation rates exceeding 30% per class and samples with over 20% missing values were systematically excluded from the cfDNA dataset. For the remaining missing values, we adopted a zero-filling approach instead of imputation to prevent potential biases, particularly in minority classes where sparse data could render imputation unreliable and potentially compromise sample independence. The processed dataset consisted of two key components: a source domain containing 2801 DNA methylation samples representing 91 distinct classes (82 cancer types and 9 healthy control types, Supplementary Table 1) and a target domain comprising 470 cfDNA methylation samples across 10 classes (nine cancer types and healthy controls, Table 1). To further refine feature selection, we employed a random forest algorithm to rank all probes by variable importance measures, initially identifying the top 10 000 CpG sites. These candidate sites were then cross-referenced with the preprocessed cfDNA methylation data, retaining only those with $\leq $30% missing values across all samples. Ultimately, 6585 CpG sites were consistently retained as the intersection of these steps: (1) initial removal of low-quality sites/samples during preprocessing, (2) zero-filling of residual missing values, and (3) algorithmic selection of high-importance sites with minimal missing data. These rigorously curated CpG sites formed the input features for the model.

**Table 1:** Overview of the cfDNA methylation datasets used in this study

**Data set**	**Data description**	**Sample size**	**Source**
PRAD	prostate cancer	181	CFEA
BRCA_1	breast cancer	123	GSE207998
BRCA_2	breast cancer	3	CFEA
HC_1	healthy control	38	CFEA
HC_2	healthy control	22	GSE129374
HC_3	healthy control	22	GSE186888
LIHC	liver hepatocellular carcinoma	22	GSE129374
Sepsis	sepsis	22	CFEA
MTLE	mesial temporal lobe epilepsy	12	GSE208758
PITR	pancreatic islet transplant recipient	9	CFEA
COAD	colon adenocarcinoma	8	CFEA
CRC	colorectal cancer	4	CFEA
LUAD	lung adenocarcinoma	4	CFEA
Total	-	470	-

### Performance metrics for robust classification evaluation

To assess its classification performance in cancer diagnostics, cfMethylPre employs the **MCC** and the **F1 score**, two metrics specifically suited for imbalanced datasets, which are frequently encountered in medical diagnostics.

The **MCC** is a robust metric that integrates true positives (TP), true negatives (TN), false positives (FP), and false negatives (FN) to provide a balanced evaluation of model performance, even in the presence of skewed class distributions. By incorporating all components of the confusion matrix, MCC effectively captures both sensitivity and specificity, making it well-suited for scenarios where the consequences of false positives and false negatives differ significantly. This holistic approach ensures a reliable assessment of a model’s predictive capability. MCC is valuable for imbalanced datasets, as it delivers a comprehensive evaluation across all classes, offering a balanced measure of model quality. Unlike accuracy or F1-score, which may be skewed by overrepresented classes, MCC mitigates bias in datasets with significant class imbalances, such as those where cancer types like BRCA and PRAD predominate.

### Visualization and enrichment analysis

To investigate the structure of sample embeddings learned under various feature scenarios, we employed Uniform Manifold Approximation and Projection (UMAP), a powerful dimensionality reduction technique that effectively preserves both local and global data structures [34]. We implemented UMAP using the Python package umap-learn (version 0.5.4).

To elucidate the biological significance of the genes identified in our study, we conducted Gene Ontology (GO) enrichment analysis using the R package clusterProfiler (version 4.16.0) [35]. This analysis aimed to identify significantly enriched biological processes, molecular functions, and cellular components associated with the gene set. GO annotations were obtained from established databases [36], and enrichment was assessed using a hypergeometric test with false discovery rate correction to account for multiple testing. GO terms with an adjusted $P$-value below.05 were deemed significantly enriched.

### Biological experiments

#### Cell lines and cell culture

The breast cancer cell lines $MCF-7$ and $MDA-MB-231$ were obtained from the Cell Bank of the Chinese Academy of Sciences in Shanghai, China, where they underwent authentication prior to use. These cell lines were cultured in Dulbecco’s Modified Eagle Medium (DMEM) supplied by Gibco, supplemented with 10% fetal bovine serum (FBS) and 1% penicillin/streptomycin solution. Culturing was conducted in a humidified incubator maintained at 37$^\circ $C with a 5% $CO_{2}$ atmosphere. The lentiviral plasmids $pLVX-Puro-PCDHA10-FLAG$, $pLVX-Puro-PRICKLE2-FLAG$, $pLVX-Puro-PCDH10-FLAG$, and $pLVX-Puro-PRTG-FLAG$ were acquired from GENTLEGEN Co., Ltd., CN. These lentiviral plasmids were packaged into a lentivirus for infection of $MCF-7$ and $MDA-MB-231$ cell lines, resulting in the generation of stable expression cell lines through puromycin selection.

#### Western bolt

For protein extraction, cells were lysed, and the lysates were then subjected to Western blot analysis. The procedure employed primary antibodies against the Flag-tag (F3165, Sigma, at a 1:1000 dilution) and GAPDH (Ab181602, Abcam, at a 1:10000 dilution) to serve as a loading control. Detection was facilitated using a Goat anti-Mouse/Rabbit IgG (H+L) HRP-conjugated secondary antibody (BS13278, Bioworlde, at a 1:10000 dilution).

#### Cell counting kit-8 assay

Cell proliferation was assessed using the cell counting kit-8 (CCK8) assay (HY-K0301, MedChemExpress, China), following the manufacturer’s protocol. Cells exhibiting stable gene expression were plated in 96-well plates at predetermined time points (0, 24, 48, and 96 h). To each well, 10 $\mu L$ of CCK8 solution was added, followed by incubation for an additional hour. Absorbance at 450 nm was measured using a Varioskan microplate spectrophotometer (Thermo Fisher Scientific). Data collected in triplicate for each time point were analyzed to determine trends in cell proliferation over the designated periods.

#### Transwell invasion assay

The invasive potential of $MCF-7$ and $MDA-MB-231$ cell lines, genetically modified to overexpress PCDHA10, PRICKLE2, PCDH10, and PRTG, was assessed through transwell invasion assays. The membranes of the transwell chambers (MCEP24H48, Millipore) were coated with a Matrigel solution (356234, BD Biosciences) at a $1:8$ dilution from a concentration of $50 mg/L$, followed by overnight drying at 4$^\circ $C. Cells were seeded at a density of $1\times 10^{5}$ per well in the upper chamber with 100 $\mu L$ of serum-free medium, while the lower chamber was filled with 500 $\mu L$ of medium containing 10% FBS to serve as a chemoattractant. After a 48-h incubation period, cells that did not invade through the membrane were gently removed. Invading cells were fixed with 4% paraformaldehyde for 30 min and then stained with 0.5% crystal violet for 30 min at room temperature. Quantitative analysis of invaded cells was conducted using an Olympus inverted microscope, accompanied by image processing software for precise cell counting. This methodology facilitates an accurate assessment of the effects of overexpressing PCDHA10, PRICKLE2, PCDH10, and PRTG on the invasion capabilities of breast cancer cells, offering critical insights into their potential role in the metastatic behavior of these cells.

## Results and discussion

### Overview of cfMethylPre framework

We developed cfMethylPre, a deep learning and transfer learning framework, to predict multiple cancers using cfDNA methylation data. To establish the model, we first collected bulk DNA methylation samples from multiple studies, resulting in a dataset comprising 2801 samples across 82 central nervous system cancer classes and nine control tissue classes. These sub-cancer types span 13 major cancer types. Additionally, we curated cfDNA methylation data from various studies, yielding a dataset of 470 samples covering nine cancer types and normal controls. The distribution of samples across these datasets is shown in Fig. 1a.

The cfMethylPre framework employs a transfer learning strategy to leverage the wealth of information in bulk DNA methylation data while adapting to the unique characteristics of cfDNA. Initially, the model is trained on the bulk DNA methylation dataset using an integrated cancer cohort of 2801 samples. Subsequently, the model is fine-tuned on the cfDNA methylation dataset by freezing specific network layers to retain learned features while adapting to the cfDNA-specific data. This transfer learning process, illustrated in Fig. 1b, enables the model to generalize effectively to cfDNA methylation while minimizing overfitting on the smaller cfDNA cohort.

The deep learning backbone of cfMethylPre is based on a ResNet architecture, designed to handle complex, high-dimensional methylation data. Beyond standard methylation profiles, the framework incorporates PPE generated using the pretrained large language model, as shown in Fig. 1c. These embeddings capture DNA sequence information around the methylation sites, enhancing the model’s ability to integrate sequence-specific patterns into cancer predictions. The sequence encoding process for individual sites is illustrated in Fig. 1d, where the sequence of CpG sites is processed using ESM-2 to obtain initial embeddings, which are then reduced to 128 dimensions via PCA to generate the final PPE used.

To further enhance its versatility, cfMethylPre includes an optional OpenMax layer, which extends the model’s capability beyond conventional softmax outputs. This layer enables the recognition of both known cancer types and potential novel cancer subtypes, ensuring robust performance even in the presence of previously unseen data.

### Performance evaluation of cfMethylPre

**Figure 2:**
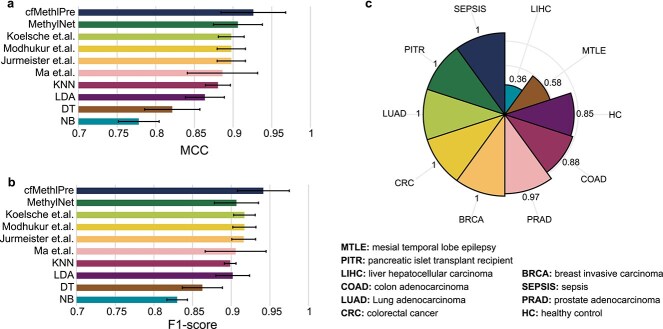
Performance evaluation of cfMethylPre: (a) MCC comparison; (b) F1-score comparison; (c) accuracy in predicting unknown classes.

We evaluated cfMethylPre using a five-fold cross-validation strategy and assessed its performance with five metrics: weighted MCC, weighted F1-score, accuracy, precision, and recall. Given the imbalanced nature of the dataset, we focused primarily on the MCC and F1-score as they provide a more balanced evaluation of the model’s performance. On the test dataset, cfMethylPre achieved a weighted MCC of 0.926 and a weighted F1-score of 0.942, with precision and recall values of 0.944 and 0.945, respectively. These results highlight cfMethylPre’s robust predictive capabilities and its ability to handle complex cfDNA methylation data effectively. We compared cfMethylPre with nine existing algorithms, including deep learning-based models such as MethylNet [23] and various machine learning approaches. These included random forest-based methods (e.g. Koelsche *et al*. [37]), XGBoost-based approaches (e.g. Ma *et al*. [38]), and SVM-based methods (e.g. Modhukur *et al*. [24]). Additionally, we evaluated cfMethylPre against four baseline models: k-nearest neighbors (KNN), linear discriminant analysis (LDA), decision tree (DT), and naïve Bayes (NB). Supplementary Table 2 summarizes the performance of all methods, while Fig. 2a and b illustrate the MCC and F1-score.

cfMethylPre demonstrated superior performance across all metrics compared with existing methods. Specifically, it achieved the highest MCC of 0.926, surpassing MethylNet (0.906) and Koelsche *et al*. (0.898), showcasing its ability to capture intricate patterns in high-dimensional methylation data. For the F1-score, cfMethylPre reached 0.942, significantly outperforming the next-best model, Koelsche *et al*. (0.917), reflecting its excellent balance between precision and recall. In contrast, traditional methods such as KNN, LDA, decision trees (DT), and Naïve Bayes (NB) performed markedly worse, with MCC values ranging from 0.821 (DT) to 0.778 (NB), underscoring their limitations in handling the complexity of cfDNA methylation data. These findings show cfMethylPre as an accurate framework for multi-cancer prediction using cfDNA methylation data, offering a significant advancement over existing methodologies.

Identifying unknown cancer types is critical for the clinical application of cancer diagnosis. To evaluate cfMethylPre’s ability to recognize unknown classes, we performed an experiment where each cancer type or healthy control group was masked one at a time. The remaining classes were used to train the model. After training, the model was tasked with predicting the labels of the masked samples as a new unknown class. The accuracy of identifying the masked samples as an unknown class was calculated to assess the model’s effectiveness. The results are presented in Fig. 2c. For five cancer types (i.e. SEPSIS, LUAD, PITR, CRC, and BRCA), the masked samples were perfectly identified as a new class, showcasing cfMethylPre’s capability to detect novel cancer types. For PRAD, COAD, and HC (healthy controls), the model achieved reasonable accuracy, with scores of 0.970, 0.875, and 0.850. However, for MTLE and LIHC, the accuracy was lower, suggesting that their cfDNA methylation profiles may not exhibit distinctive patterns that are as easily separable from other classes. These findings highlight cfMethylPre’s robust ability to generalize beyond known cancer types, enabling the identification of novel or uncharacterized cancers.

To further validate the generalization ability of cfMethylPre, we tested the trained model on an independent dataset (GSE214344), which includes cfDNA methylation data of seven breast cancer cases and five healthy controls [39]. On this dataset, cfMethylPre achieved an MCC of 0.700 and an F1-score of 0.857, demonstrating its capability to generalize to unseen data.

To evaluate the effectiveness of the backbone structure and transfer learning strategy in cfMethylPre, we compared two architectures, ResNet18 and ResNet101, each with five freezing strategies: (1) freezing all layers and training only the classification head (fully frozen backbone), (2) freezing the first three blocks, (3) freezing the first two blocks, (4) freezing the first block, and (5) training all layers without freezing. For the fully frozen backbone, all convolutional layers were fixed, with only the classification head trained. For freezing more layers, we progressively fixed additional blocks (e.g. the first three blocks) while fine-tuning the remaining layers. Our findings confirm that our chosen strategy, using ResNet101 with the first two blocks frozen while fine-tuning the latter two, is effective, achieving the best balance between leveraging prior knowledge from DNA methylation data and adapting to cfDNA-specific patterns, as detailed in Supplementary Table 3. Furthermore, We analyzed the performance of CNN and ResNet as backbone models, paired with DNABERT, DNABERT-2, and ESM-2 for sequence embedding. The results showed that the ResNet and ESM-2 combination yielded the best performance. Detailed findings are presented in Supplementary Table 4.

To address the class imbalance in our cfDNA cohort, where breast and prostate cancer samples were overrepresented compared with other classes, we explored two additional strategies beyond transfer learning and MCC evaluation: class weighting and oversampling. In the class weighting approach, we adjusted the loss function by assigning weights to each class, calculated as the total number of samples divided by the number of samples in a specific class, to enhance the influence of minority classes. Additionally, we applied Synthetic Minority Oversampling Technique to oversample minority classes. While these methods improved the accuracy of minority classes, they led to a decrease in overall accuracy and were therefore not included in subsequent experiments. Detailed results of these experiments are presented in Supplementary Table 5.

To evaluate computational efficiency, we measured the running time of our method alongside other approaches on the same platform (NVIDIA RTX 4090 GPU, 24GB memory). The training process required about 2 h per fold over 250 epochs, while inference averaged 2.317 s per sample. Compared with traditional machine learning methods on identical hardware, our pipeline exhibits longer training times due to the inherent computational demands of deep learning and CpG-site-level data augmentation. However, this trade-off delivers significantly higher accuracy, which is critical for real-world applications. Benchmarking results for comparative methods are provided in Supplementary Table 6.

### Effects of integrating the DNA sequence information with cfDNA methylation profiles

**Figure 3:**
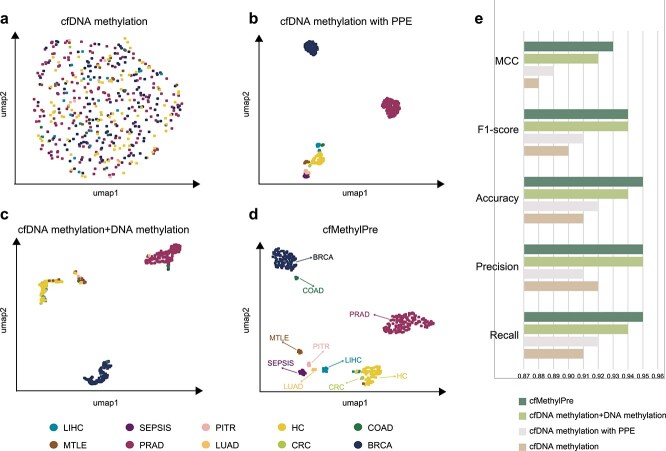
Effects of integrating the DNA sequence information and applying transfer learning. (a) UMAP visualization indicating clustering performance using only cfDNA methylation data. (b) Using only probe sequence information with cfDNA methylation data. (c) Using cfDNA methylation data after pretraining with the bulk DNA methylation data. (d) Performance of cfMethylPre. (e) Classification metrics.

To evaluate the contribution of DNA sequence information and transfer learning to the performance of cfMethylPre, we designed experiments using different feature combinations: (1) cfDNA methylation only, (2) cfDNA methylation with PPE generated by the large language model, (3) transfer learning using bulk DNA methylation followed by cfDNA methylation fine-tuning, and (4) the full cfMethylPre framework combining transfer learning, cfDNA methylation, and PPE features. The sample embeddings learned under each feature scenario were visualized using UMAP, with the resulting projections shown in Figs 3a-d. The clustering and classification performances were subsequently quantified using MCC, F1-score, accuracy, precision, and recall metrics (Fig. 3e). Detailed results for these metrics are provided in Supplementary Table 7.

As shown in Fig. 3a, when using only cfDNA methylation data, the samples could not be stratified into distinct groups. The UMAP plot showed no meaningful clustering, indicating limited discriminatory power. Integrating DNA sequence information with cfDNA methylation profiles (Fig. 3b) significantly improved clustering. The UMAP plot revealed three clusters corresponding to breast cancer (BRCA), prostate cancer (PRAD), and other classes. However, the third cluster, representing healthy controls and minor cancer types, showed overlap, likely due to the smaller sample sizes of these groups. The MCC and F1-score improved to 0.890 and 0.910, respectively, demonstrating the added value of PPE features for capturing sequence-based context around methylation sites. Incorporating transfer learning by pretraining the model on bulk DNA methylation data and fine-tuning it with cfDNA methylation samples (Fig. 3c) further enhanced clustering performance. Healthy controls showed a trend toward separating from minor cancer types, although some cancer samples were still mixed with controls. The MCC and F1-score increased to 0.920 and 0.940, validating the efficacy of transfer learning in leveraging bulk methylation data to improve cfDNA predictions.

The full cfMethylPre model, integrating transfer learning, cfDNA methylation, and PPE features (Fig. 3d), achieved the best clustering and classification results. Nearly all sample groups were clearly separated in the UMAP plot, highlighting the synergistic effect of combining sequence-based features and transfer learning. The MCC and F1-score peaked at 0.930 and 0.940, respectively, marking the highest performance among all scenarios. Comparing Fig. 3d with Fig. 3c underscores the added benefit of incorporating PPE features, while comparisons between Figs 3b and d highlight the transformative impact of transfer learning. These results conclusively demonstrate the critical role of DNA sequence integration and transfer learning in enhancing the performance and generalizability of cfMethylPre for multi-cancer prediction using cfDNA methylation data.

### Interpretability and biological insights in breast cancer prediction

To evaluate the interpretability of cfMethylPre in cancer prediction, we applied the model to distinguish between breast cancer patients and healthy individuals, achieving an exceptional accuracy of 0.977. To gain biological insights into the underlying features driving the model’s predictions, we conducted a gradient-based feature attribution analysis. This approach ranked the contribution of individual methylation sites to the model’s classification power. The methylation sites were subsequently mapped to corresponding genes using gene annotation files specific to the sequencing technology. The top 100 probes, corresponding to 68 unique genes, with the highest contributions to the model’s predictions were selected for further analysis and are detailed in Supplementary Table 8.

**Figure 4:**
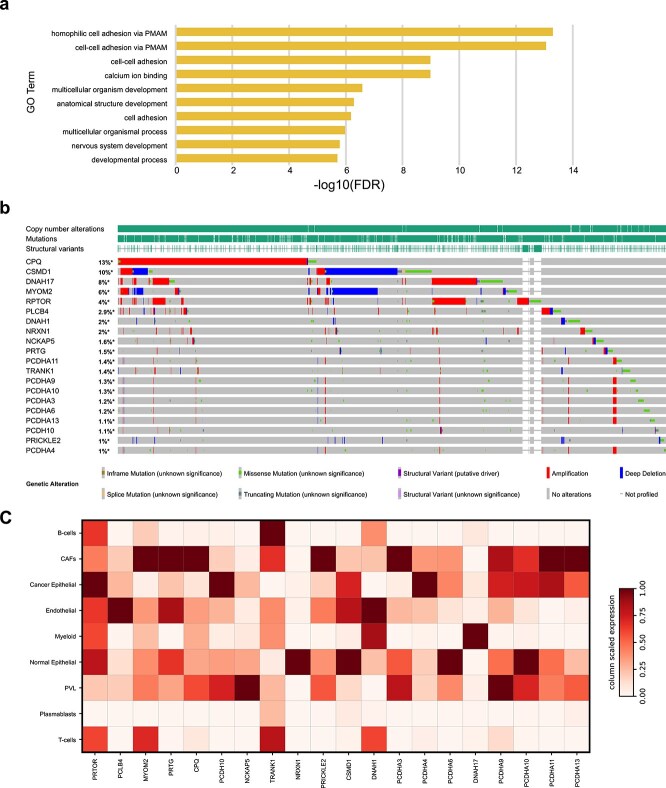
Biological insights in breast cancer prediction. (a) Top 10 enriched GO biological processes from model interpretability-derived genes. (b) Top 20 genes with the highest somatic mutation rates among screened genes. (c) Normalized expression of the 20 genes in single-cell RNA-seq data.

We performed GO enrichment analysis on the 68 genes, and the top 10 significantly enriched biological processes are shown in Fig. 4a. Detailed results of the enrichment analysis are provided in Supplementary Table 9. These processes are closely associated with the development and progression of breast cancer. For example, cell adhesion processes (homophilic and general adhesion) are crucial for maintaining tissue architecture, and their dysregulation is a hallmark of cancer metastasis. Altered adhesion molecule expression has been observed in breast cancer, promoting tumor cell migration and invasion [40, 41]. Additionally, multicellular organismal processes play an important role in promoting tumor microenvironment remodeling and advancing cancer progression. By mediating intercellular interactions and preserving tissue architecture, these processes create a supportive niche for cancer cells [42]. Calcium ion binding is vital in intracellular signaling pathways, including those involved in tumor cell proliferation and migration. Dysregulated calcium signaling has been linked to breast cancer progression [43]. Furthermore, developmental processes, particularly the aberrant activation of signaling pathways such as Notch, Wnt, and Hedgehog, play a critical role in breast cancer progression by driving tumor growth, cellular proliferation, and metastatic potential [44]. Consistent with our approach for breast cancer, we analyzed the top 100 high-impact loci associated with prostate cancer and identified key pathways strongly correlated with tumor progression. These include homophilic and general cell adhesion processes, multicellular organismal processes, and calcium ion binding, underscoring the critical role of the mapped genes in prostate cancer pathogenesis [40–43]. Detailed results are provided in Supplementary Table 10.

To further elucidate the pathogenic mechanisms underlying breast cancer and validate the significance of the genes identified by cfMethylPre, we analyzed their somatic mutation rates in The Cancer Genome Atlas (TCGA) and Memorial Sloan Kettering (MSK) breast cancer datasets available through cBioPortal [45–47]. Among the 68 genes, we selected 20 genes with the highest mutation rates for detailed analysis, as illustrated in Fig. 4b. Several of these 20 genes have well-documented roles in breast cancer. *RPTOR* has been implicated in mediating resistance to *EGFR* inhibitors in triple-negative breast cancer, with targeting the mTORC1 pathway emerging as a potential therapeutic strategy [48]. *MYOM2*, frequently downregulated in breast cancer, is proposed to function as a tumor suppressor, with its reduced expression associated with aggressive tumor phenotypes [49]. *CPQ* has been found to be significantly upregulated in breast cancer, suggesting its potential role in cancer progression [50]. *PCDH10* is recognized as a pan-cancer tumor suppressor, and its role in breast cancer further emphasizes its broad relevance across multiple malignancies [50, 51]. *CSMD1* functions as a tumor suppressor, with reduced expression linked to poor prognosis; its re-expression has been shown to inhibit critical malignant behaviors, including migration, invasion, and metastasis [52]. Additionally, *PCDHA9* has been identified as a tumor suppressor, with low expression rendering breast cancer cells selectively sensitive to TAS-102 treatment, indicating its therapeutic potential [53].

To uncover novel gene targets, we analyzed the expression levels of these genes in single-cell RNA sequencing data from a breast cancer dataset [54]. The cell type distribution of this single-cell dataset is presented in Supplementary Figure 2. The single-cell data were normalized using a min-max scaling approach, with expression values adjusted between 0 and 1 (Fig. 4c). Detailed expression values are provided in Supplementary Table 11. We also performed survival analysis using the KMplot platform, and conducted differential expression gene (DEG) analysis based on the breast cancer RNA-seq dataset from TCGA [55]. The results are summarized in Supplementary Table 12. Among the 20 genes, we identified novel gene targets that met the following criteria: normalized gene expression level in cancer epithelial cells $\geq $0.15, the log-rank $P$-value of survival analysis $\leq $.05, and the DEG $P$-value $\leq $.05. Four genes were identified: *PRTG*, *PRICKLE2*, *PCDHA6*, and *PCDHA10*. Notably, *PCDHA6* and *PCDHA10*, members of the Protocadherin Alpha family, exhibit co-expression patterns. *PCDHA10* was prioritized for experimental validation due to its more significant differential expression (DEG) $P$-value.

**Figure 5:**
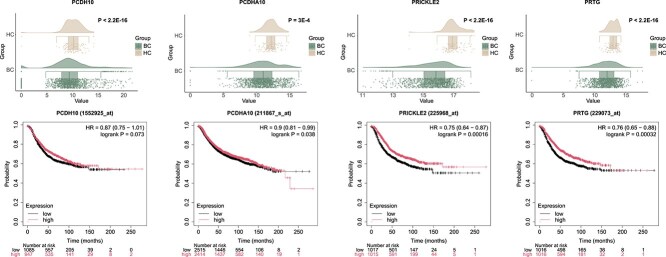
Differential expression of *PCDH10*, *PCDHA10*, *PRICKLE2*, and *PRTG* in TCGA-BRCA (cancer vs. adjacent tissue) and survival analysis stratified by median expression, with BC and HC denoting breast cancer and healthy controls.

Based on these findings, we selected the genes *PRTG*, *PRICKLE2*, *PCDHA10*, and *PCDH10* for subsequent biological experiments. *PCDHA10*, *PRTG*, and *PRICKLE2* were identified as novel breast cancer-associated candidates based on stringent criteria, including somatic mutation rates, RNA expression, prognostic significance, and differential expression. *PCDH10*, a well-established pan-cancer tumor suppressor, was included as a positive control. The expression profiles of these four genes in the single-cell RNA sequencing data are presented in Supplementary Figure 3. Among these, *PCDH10* was included as a positive control due to its well-established role as a pan-cancer tumor suppressor. The expression levels of these four genes, along with their Kaplan–Meier survival curves, are shown in Fig. 5, underscoring their potential clinical relevance in breast cancer prognosis. Survival curves for the remaining genes are available in Supplementary Figure 4.

### Functional characterization of identified genes in breast cancer cell lines

**Figure 6:**
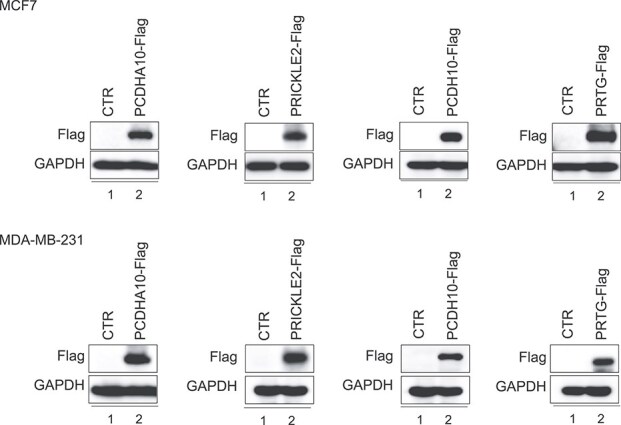
Successful construction of *PCDHA10*, *PRICKLE2*, *PCDH10*, and *PRTG* overexpression cell lines.

To investigate the functional roles of the identified genes—$PCDHA10$, $PRICKLE2$, $PCDH10$, and $PRTG$—in breast cancer progression, we evaluated their effects on cell proliferation and invasion in $MCF-7$ and $MDA-MB-231$ breast cancer cell lines. These genes were overexpressed in both cell lines through the establishment of stable cell lines, and successful overexpression was confirmed via Western blot analysis (Fig. 6).

**Figure 7:**
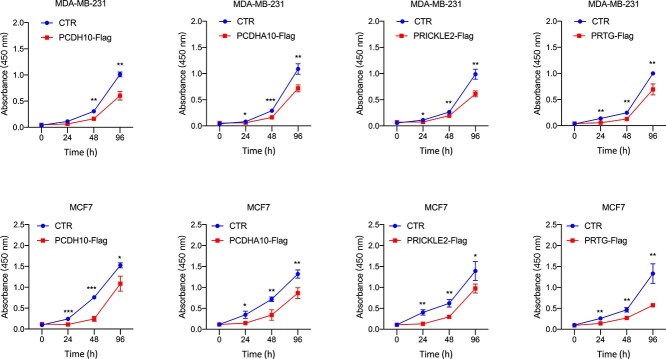
Overexpression of *PCDHA10*, *PRICKLE2*, *PCDH10*, and *PRTG* significantly decreased the proliferation of $MCF-7$ and $MDA-MB-231$ cells. To assess cell growth, we conducted the CCK8 assay on both the negative control (CTR) and the overexpression (Flag) groups of $MCF-7$ and $MDA-MB-231$ cells at 0, 24, 48, and 96 h. Statistical significance is indicated by *($P$-value $<.05$), ** ($P$-value $<.01$), and *** ($P$-value $<.001$).

The proliferation rates of $MCF-7$ and $MDA-MB-231$ cells overexpressing *PCDHA10*, *PRICKLE2*, and *PRTG* were significantly reduced, as illustrated in Fig. 7. These results underscore the inhibitory effects of these genes on breast cancer cell growth, highlighting their potential tumor-suppressive roles. *PCDH10*, included as a positive control, also demonstrated a notable reduction in proliferation, consistent with its established function as a pan-cancer tumor suppressor.

**Figure 8:**
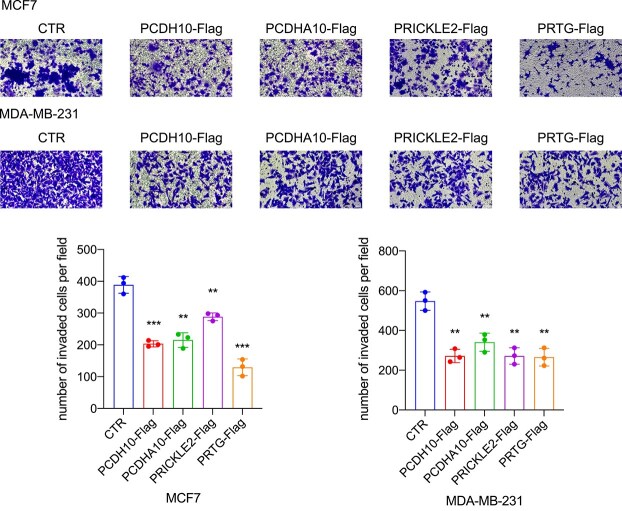
Overexpression of *PCDHA10*, *PRICKLE2*, *PCDH10*, and *PRTG* decreased the invasion capacities of $MCF-7$ and $MDA-MB-231$ cells. Invaded cells, stained and enumerated in at least three distinct microscopic fields, were documented using a 200x magnification microscope. Statistical significance is indicated by ** ($P$-value $<.01$) and *** ($P$-value $<.001$).

The invasive abilities of $MCF-7$ and $MDA-MB-231$ cells were assessed using transwell invasion assays. Overexpression of *PCDHA10*, *PRICKLE2*, *PCDH10*, and *PRTG* led to a marked decrease in invasion capacity, as shown in Fig. 8. This indicates the potential of these genes to hinder the metastatic behavior of breast cancer cells.

Together, these experiments validate the tumor-suppressive effects of *PCDHA10*, *PRICKLE2*, and *PRTG* in breast cancer and further confirm the role of *PCDH10* as a critical regulator of cancer cell proliferation and invasion. These findings provide experimental evidence for the clinical relevance of these genes and their potential as therapeutic targets in breast cancer treatment.

## Conclusion

This study introduces cfMethylPre, a novel transfer learning framework tailored to leverage cfDNA methylation data for cancer detection. By integrating pretrained sequence embeddings, deep learning, and domain adaptation, cfMethylPre addresses the challenges of limited cfDNA sample sizes and high-dimensional methylation data, providing a robust, accurate, and interpretable solution for cancer diagnostics. The framework’s ability to utilize pretrained knowledge from extensive DNA methylation datasets and fine-tune it for cfDNA-specific characteristics has demonstrated superior predictive accuracy and generalizability across multiple cancer types.

cfMethylPre outperforms existing methods in both prediction accuracy and robustness, particularly when applied to small cfDNA datasets. Furthermore, its interpretability enables the identification of critical cancer-associated genes, with experimental validation uncovering three novel genes—*PCDHA10*, *PRICKLE2*, and *PRTG*—that exhibit inhibitory effects on breast cancer cell proliferation and migration. These findings highlight cfMethylPre’s potential to contribute not only to diagnostic accuracy but also to biological discovery and therapeutic development.

Future work will focus on expanding cfMethylPre’s applicability to a wider range of cancer types and exploring its potential for longitudinal monitoring of disease progression and treatment responses. By addressing the limitations of existing cfDNA-based diagnostic tools, cfMethylPre sets the stage for liquid biopsy technologies to become integral components of precision oncology, advancing early detection, personalized treatment, and improved clinical outcomes.

**Key Points**

- This work presents cfMethylPre, a deep transfer learning framework for cancer detection using cfDNA methylation data, achieving high predictive accuracy while enabling the identification of key cancer-associated genes.
- cfMethylPre leverages large language model pretrained DNA sequence embeddings and integrates them with methylation profiles to enhance feature representation. The deep transfer learning strategy involves pretraining on bulk DNA methylation data encompassing 2801 samples across 82 cancer types and normal controls, followed by fine-tuning with 470 cfDNA methylation samples.
- Using cfMethylPre, we identified and validated three novel genes—*PCDHA10*, *PRICKLE2*, and *PRTG*—that exhibit inhibitory effects on breast cancer cell proliferation and migration, highlighting its potential in precision oncology.

## Supplementary Material

Supplementary_material_done_bbaf303

## Data Availability

The code is available at https://github.com/zxc-CCC/cfMethylPre.
