# Supplementary material for: cfMethylPre: deep transfer learning enhances cancer detection based on circulating cell-free DNA methylation profiling
Source: Brief Bioinform. 2025 Jun 29;26(3):bbaf303. doi: 10.1093/bib/bbaf303 (PMC12206449; doi:10.1093/bib/bbaf303)
Supplement: Supplementary_material_done_bbaf303 [file supplementary_material_done_bbaf303.docx]

**Supplementary Material**

**[Supplementary Table 1.](#_Toc3774)** [Summary of DNA Methylation Datasets. 1](#_Toc3774)

**[Supplementary Table 2.](#_Toc23494)** [Summary of evaluation results of cfMethylPre and compared methods. The average values of five metrics are shown through five-fold cross-validation (best results in bold). 4](#_Toc23494)

**[Supplementary Table 3.](#_Toc30197)** [Performance comparison of ResNet18 and ResNet101 architectures with five transfer learning freezing strategies in cfMethylPre. Values represent five-metric averages from five-fold cross-validation (best results in bold). 5](#_Toc30197)

**[Supplementary Table 4.](#_Toc1219)** [Summary of evaluation results using different language models and backbone models in cfMethylPre. Values are average metrics from five-fold cross-validation (best results in bold). 6](#_Toc1219)

**[Supplementary Table 5.](#_Toc23733)** [Summary of sample imbalance evaluation results using different strategies. Average values of five metrics are reported from five-fold cross-validation (best results in bold). 7](#_Toc23733)

**[Supplementary Table 6.](#_Toc12926)** [Running time benchmark of cfMethylPre and compared methods. 8](#_Toc12926)

**[Supplementary Table 7.](#_Toc6197)** [Performance metrics of models in ablation experiments.Values represent five-metric averages from five-fold cross-validation (best results in bold). 9](#_Toc6197)

**[Supplementary Table 8.](#_Toc29194)** [Top 100 probes mapped to 68 genes with corresponding impact scores in breast cancer. 10](#_Toc29194)

**[Supplementary Table 9.](#_Toc17168)** [Enrichment analysis of genes mapped by the top 100 probes for breast cancer. 17](#_Toc17168)

**[Supplementary Table 10.](#_Toc30238)** [Enrichment analysis of genes mapped by the top 100 probes for prostate cancer. 18](#_Toc30238)

**[Supplementary Table 11.](#_Toc25983)** [Standardized expression values of top 100 probes mapped to 68 genes across major cell types in breast cancer. 19](#_Toc25983)

**[Supplementary Table 12.](#_Toc19694)** [Summary of the top 20 genes ranked by model explainable ability, including somatic mutation rates, normalized expression levels in cancer single cells, and significance values from survival and differential expression analyses. 20](#_Toc19694)

**[Supplementary Figure 1.](#_Toc2448)** [Framework of the full ResNet architecture with integrated PPE module. 21](#_Toc2448)

**[Supplementary Figure 2.](#_Toc4451)** [Uniform manifold approximation and projection map (UMAP) of 100064 cells color-coded for the indicated cell type. 22](#_Toc4451)

**[Supplementary Figure 3.](#_Toc31302)** [Expression of](#_Toc31302) *[PRICKLE2](#_Toc31302)*[,](#_Toc31302) *[PCDH10](#_Toc31302)*[,](#_Toc31302) *[PCDHA10](#_Toc31302)*[, and](#_Toc31302) *[PRTG](#_Toc31302)* [genes in single-cell dataset. 23](#_Toc31302)

**[Supplementary Figure 4.](#_Toc18274)** [Kaplan–Meier analysis of relapse-free survival (RFS) for the top 20 genes with the highest mutation rates among the top 100 probes mapped to high-impact genes. 24](#_Toc18274)

**Supplementary Table 1.** Summary of DNA Methylation Datasets.

| **Primary Category** | **Category Size** | **Subtype** | **Subtype Size** |
| --- | --- | --- | --- |
| Embryonal | 607 | 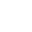ETMR | 43 |
|  |  | MB, WNT | 39 |
|  |  | MB, G3 | 77 |
|  |  | MB, G4 | 138 |
|  |  | MB, SHH CHL AD | 84 |
|  |  | MB, SHH INF | 52 |
|  |  | ATRT, MYC | 29 |
|  |  | ATRT, SHH | 46 |
|  |  | ATRT, TYR | 37 |
|  |  | CNS NB, FOXR2 | 39 |
|  |  | HGNET, BCOR | 23 |
| Glioblastoma | 425 | DMG, K27 | 78 |
|  |  | GBM, G34 | 41 |
|  |  | GBM, MES | 56 |
|  |  | GBM, MID | 14 |
|  |  | GBM, MYCN | 16 |
|  |  | GBM, RTK I | 64 |
|  |  | GBM, RTK II | 143 |
|  |  | GBM, RTK III | 13 |
| Glio-neuronal | 198 | CN | 21 |
|  |  | DLGNT | 8 |
|  |  | LIPN | 10 |
|  |  | LGG, DIG/DIA | 8 |
|  |  | LGG, DNT | 44 |
|  |  | RETB | 19 |
|  |  | ENB, A | 23 |
|  |  | ENB, B | 16 |
|  |  | PGG, nC | 19 |
|  |  | LGG, GG | 21 |
|  |  | LGG, RGNT | 9 |
| Sella | 169 | CPH, ADM | 25 |
|  |  | CPH, PAP | 20 |
|  |  | PITAD, ACTH | 18 |
|  |  | PITAD, FSH LH | 21 |
|  |  | PITAD, PRL | 8 |
|  |  | PITUI, SCO, GCT | 29 |
|  |  | PITAD, STH DNS B | 12 |
|  |  | PITAD, STH SPA | 17 |
|  |  | PITAD, STH DNS A | 9 |
|  |  | PITAD, TSH | 10 |
| Gliona IDH | 204 | A IDH | 78 |
| **Primary Category** | **Category Size** | **Subtype** | **Subtype Size** |
| Gliona IDH | 204 | A IDH, HG | 46 |
|  |  | O IDH | 80 |
| Ependymal | 343 | EPN, RELA | 70 |
|  |  | EPN, YAP | 11 |
|  |  | EPN, PF A | 91 |
|  |  | EPN, PF B | 51 |
|  |  | EPN, SPINE | 27 |
|  |  | EPN, MPE | 28 |
|  |  | SUBEPN, PF | 37 |
|  |  | SUBEPN, SPINE | 9 |
|  |  | SUBEPN, ST | 19 |
| Other glioma | 327 | CHGL | 12 |
|  |  | LGG, SEGA | 21 |
|  |  | LGG, PA MID | 38 |
|  |  | LGG, PA/GG ST | 24 |
|  |  | LGG, PA PF | 114 |
|  |  | PXA | 44 |
|  |  | HGNET, MN1 | 21 |
|  |  | IHG | 10 |
|  |  | LGG, MYB | 22 |
|  |  | ANA PA | 21 |
| Nerve | 31 | SCHW | 23 |
|  |  | SCHW, MEL | 8 |
| Pineal | 80 | PTPR, A | 8 |
|  |  | PTPR, B | 22 |
|  |  | PIN T, PB A | 9 |
|  |  | PIN T, PB B | 22 |
|  |  | PIN T, PPT | 19 |
| Mesenchymal | 167 | CHORDM | 9 |
|  |  | EWS | 14 |
|  |  | HMB | 25 |
|  |  | MNG | 90 |
|  |  | SFT HMPC | 16 |
|  |  | EFT, CIC | 13 |
| Melanocytic | 27 | MELAN | 12 |
|  |  | MELCYT | 15 |
| Plexus | 83 | PLEX, AD | 22 |
|  |  | PLEX, PED A | 15 |
|  |  | PLEX, PED B | 46 |
| Haematopoietic | 21 | LYMPHO | 13 |
|  |  | PLASMA | 8 |
| Control | 119 | CONTR, ADENOPIT | 9 |
|  |  | CONTR, CEBM | 8 |
| **Primary Category** | **Category Size** | **Subtype** | **Subtype Size** |
| Control | 119 | CONTR, HEMI | 13 |
|  |  | CONTR, HYPTHAL | 9 |
|  |  | CONTR, INFLAM | 24 |
|  |  | CONTR, PINEAL | 12 |
|  |  | CONTR, PONS | 12 |
|  |  | CONTR, REACT | 23 |
|  |  | CONTR, WM | 9 |

**Supplementary Table 2.** Summary of evaluation results of cfMethylPre and compared methods. The average values of five metrics are shown through five-fold cross-validation (best results in bold).

| **Method** | **MCC** | **F1-score** | **Accuracy** | **Precision** | **Recall** |
| --- | --- | --- | --- | --- | --- |
| cfMethylPre | **0.926** | **0.942** | **0.945** | **0.944** | **0.945** |
| MethylNet | 0.906 | 0.907 | 0.934 | 0.886 | 0.934 |
| Koelsche et.al. | 0.898 | 0.917 | 0.919 | 0.905 | 0.919 |
| Modhukur et.al. | 0.898 | 0.917 | 0.906 | 0.888 | 0.906 |
| Jurmeister et.al. | 0.898 | 0.916 | 0.923 | 0.919 | 0.923 |
| Ma et.al. | 0.886 | 0.906 | 0.915 | 0.907 | 0.915 |
| KNN | 0.880 | 0.899 | 0.911 | 0.903 | 0.911 |
| LDA | 0.864 | 0.902 | 0.896 | 0.926 | 0.896 |
| DT | 0.821 | 0.863 | 0.874 | 0.866 | 0.874 |
| NB | 0.778 | 0.830 | 0.830 | 0.859 | 0.830 |

**Supplementary Table 3.** Performance comparison of ResNet18 and ResNet101 architectures with five transfer learning freezing strategies in cfMethylPre. Values represent five-metric averages from five-fold cross-validation (best results in bold).

| **Method** | **MCC** | **F1-score** | **Accuracy** | **Precision** | **Recall** | **AUROC** |
| --- | --- | --- | --- | --- | --- | --- |
| ResNet18-0 | 0.773 | 0.85 | 0.871 | 0.843 | 0.857 | 0.911 |
| ResNet18-1 | 0.914 | 0.931 | 0.935 | 0.917 | 0.945 | 0.985 |
| ResNet18-2 | 0.916 | 0.932 | 0.937 | 0.919 | 0.947 | 0.984 |
| ResNet18-3 | 0.923 | 0.927 | 0.943 | 0.903 | 0.953 | 0.983 |
| ResNet18-4 | 0.895 | 0.917 | 0.922 | 0.913 | 0.922 | 0.984 |
| ResNet101-0 | 0.812 | 0.833 | 0.888 | 0.784 | 0.891 | 0.902 |
| ResNet101-1 | 0.916 | 0.923 | 0.925 | 0.919 | 0.94 | 0.982 |
| ResNet101-2 | **0.926** | **0.942** | **0.945** | **0.944** | **0.962** | **0.986** |
| ResNet101-3 | 0.919 | 0.941 | 0.943 | 0.930 | 0.953 | 0.983 |
| ResNet101-4 | 0.892 | 0.915 | 0.919 | 0.912 | 0.919 | 0.984 |

**Note**: -0: classification head only (all convolutional layers frozen); -1: last block trainable;-2: last two blocks trainable;-3: last three blocks trainable;-4: all layers trainable (no freezing).

**Supplementary Table 4.** Summary of evaluation results using different language models and backbone models in cfMethylPre. Values are average metrics from five-fold cross-validation (best results in bold).

| **Method** | **MCC** | **F1-score** | **Accuracy** | **Precision** | **Recall** | **AUROC** |
| --- | --- | --- | --- | --- | --- | --- |
| Resnet+ESM2(cfMethylPre) | **0.926** | **0.942** | **0.945** | **0.944** | **0.962** | **0.986** |
| Resnet+DNABert | 0.906 | 0.937 | 0.931 | 0.928 | 0.947 | 0.977 |
| Resnet+DNABert2 | 0.915 | 0.938 | 0.936 | 0.933 | 0.949 | 0.980 |
| CNN+ESM2 | 0.853 | 0.887 | 0.920 | 0.885 | 0.895 | 0.960 |
| CNN+DNABert | 0.825 | 0.882 | 0.910 | 0.875 | 0.885 | 0.955 |
| CNN+DNABert2 | 0.823 | 0.883 | 0.914 | 0.882 | 0.884 | 0.957 |

**Supplementary Table 5.** Summary of sample imbalance evaluation results using different strategies. Average values of five metrics are reported from five-fold cross-validation (best results in bold).

| **Method** | **MCC** | **F1-score** | **Accuracy** | **Precision** | **Recall** | **AUROC** |
| --- | --- | --- | --- | --- | --- | --- |
| cfMethylPre | **0.926** | **0.942** | **0.945** | **0.944** | **0.962** | **0.986** |
| Weighted Loss Function | 0.899 | 0.920 | 0.943 | 0.910 | 0.930 | 0.975 |
| SMOTE | 0.853 | 0.875 | 0.920 | 0.855 | 0.895 | 0.960 |

**Supplementary Table 6.** Running time benchmark of cfMethylPre and compared methods.

| **Method** | **training time** |
| --- | --- |
| cfMethylPre | 2 hours 20 minutes |
| MethylNet | 1 hours 11 minutes |
| Koelsche et.al. | 1 hours 27 minutes |
| Modhukur et.al. | 1 hours 42 minutes |
| Jurmeister et.al. | 13 minutes |
| Ma et.al. | 13 minutes |
| KNN | 15 minutes |
| LDA | 8 minutes |
| DT | 9 minutes |
| NB | 13 minutes |

**Supplementary Table 7.** Performance metrics of models in ablation experiments.Values represent five-metric averages from five-fold cross-validation (best results in bold).

| **Model** | **MCC** | **F1_score** | **Accuracy** | **Precision** | **Recall** | **AUROC** |
| --- | --- | --- | --- | --- | --- | --- |
| cfMethyPre | **0.926** | **0.942** | **0.945** | **0.944** | **0.945** | **0.986** |
| cfDNA methylation+DNA methylation | 0.921 | 0.939 | 0.940 | 0.943 | 0.936 | 0.990 |
| cfDNA methylation with PPE | 0.892 | 0.913 | 0.919 | 0.912 | 0.919 | 0.984 |
| cfDNA methylation | 0.882 | 0.905 | 0.911 | 0.909 | 0.903 | 0.951 |

**Supplementary Table 8.** Top 100 probes mapped to 68 genes with corresponding impact scores in breast cancer.

| **GeneSymbol_inner** | **Gene_pos** | **CpG** | **Score** | **GeneSymbol** | **Group** | **RelationToIsland** |
| --- | --- | --- | --- | --- | --- | --- |
| ANXA2 | chr15:60,347,151-60,397,986 | cg02072495 | 141.3515769 | ANXA2 | 5'UTR;Body | N_Shore |
| ARHGAP27P1-BPTFP1-KPNA2P3 | chr17:64,749,663-64,781,626 | cg16561266 | 236.4941893 | ARHGAP27P1 | TSS200 | Island |
|  |  | cg16561266 | 236.4941893 | ARHGAP27P1-BPTFP1-KPNA2P3 | Body | Island |
| ASIP | chr20:34,241,423-34,269,344 | cg16655240 | 58.54263191 | ASIP | 3'UTR | Island |
| BAIAP2 | chr17:81,035,151-81,117,434 | cg12475142 | 49.42468115 | BAIAP2 | Body | S_Shelf |
| C17orf107 | chr17:4,899,536-4,902,934 | cg14482741 | 81.25768517 | C17orf107 | TSS200 | Island |
| C2CD4A | chr15:62,066,977-62,070,917 | cg01383890 | 56.80178641 | C2CD4A | TSS200 | N_Shore |
| CCDC140 | chr2:222,298,147-222,305,217 | cg14559259 | 73.56552428 | CCDC140 | 5'UTR | N_Shore |
| CD40 | chr20:46,118,314-46,129,858 | cg11841529 | 60.1832522 | CD40 | TSS200 | N_Shore |
| CHRNE | chr17:4,897,771-4,903,098 | cg14482741 | 81.25768517 | CHRNE | Body | Island |
| CLDN11 | chr3:170,418,868-170,434,691 | cg17078427 | 50.98621304 | CLDN11 | Body | Island |
| COL20A1 | chr20:63,293,186-63,334,806 | cg14992000 | 151.6462031 | COL20A1 | Body | N_Shelf |
| CPQ | chr8:96,645,242-97,143,501 | cg23741863 | 69.8498562 | CPQ | 5'UTR | Island |
| CSMD1 | chr8:2,935,361-4,994,914 | cg08373605 | 15.99848376 | CSMD1 | Body | OpenSea |
| DNAH1 | chr3:52,316,319-52,400,492 | cg22084642 | 14.11695345 | DNAH1 | 5'UTR | OpenSea |
| DNAH17 | chr17:78,423,697-78,577,396 | cg05561386 | 11.83908887 | DNAH17 | Body | Island |
| DNAI2 | chr17:74,274,234-74,314,884 | cg17562433 | 54.23569572 | DNAI2 | TSS1500 | N_Shore |
| EBF2 | chr8:25,841,725-26,045,413 | cg22324567 | 115.119265 | EBF2 | Body | Island |
| FEZF2 | chr3:62,369,681-62,373,550 | cg12378187 | 75.46849717 | FEZF2 | 3'UTR | Island |
|  |  | cg20072171 | 120.3208584 | FEZF2 | Body | Island |
| FOXA2 | chr20:22,580,998-22,584,572 | cg16963144 | 56.48792126 | FOXA2 | TSS1500 | Island |
| FSCN2 | chr17:81,528,377-81,537,130 | cg16201883 | 172.8536565 | FSCN2 | 1stExon | Island |
| GAL3ST2 | chr2:241,776,822-241,804,287 | cg15346191 | 96.58144772 | GAL3ST2 | 3'UTR | Island |
| HELZ2 | chr20:63,558,086-63,574,239 | cg19513282 | 69.95750882 | HELZ2 | Body | Island |
| **GeneSymbol_inner** | **Gene_pos** | **CpG** | **Score** | **GeneSymbol** | **Group** | **RelationToIsland** |
| KCNB1 | chr20:49,363,877-49,482,668 | cg11131532 | 58.34642499 | KCNB1 | TSS200 | Island |
| LINC00606 | chr3:10,759,484-10,764,192 | cg19764594 | 75.13733357 | LINC00606 | TSS1500 | OpenSea |
| MKS1 | chr17:58,205,441-58,219,255 | cg15648345 | 56.91277285 | MKS1 | TSS1500 | S_Shore |
| MYOM2 | chr8:2,045,046-2,145,456 | cg21816685 | 100.3400821 | MYOM2 | Body | OpenSea |
| NCKAP5 | chr2:132,671,788-133,568,463 | cg14416623 | 58.36996252 | NCKAP5 | Body | OpenSea |
| NRXN1 | chr2:49,918,503-51,032,132 | cg14875171 | 50.66948109 | NRXN1 | 1stExon;Body;5'UTR | Island |
| NT5DC2 | chr3:52,524,387-52,533,857 | cg18337363 | 90.5328769 | NT5DC2 | 5'UTR;TSS1500;1stExon | N_Shore |
| OTX2 | chr14:56,799,905-56,810,479 | cg23365739 | 88.3311247 | OTX2 | 1stExon;5'UTR | N_Shore |
| PCDH10 | chr4:133,149,294-133,194,700 | cg00160440 | 64.97652487 | PCDH10 | Body | S_Shelf |
| PCDHA1 | chr5:140,786,140-141,012,347 | cg00275741 | 0.680151182 | PCDHA1 | Body | N_Shore |
|  |  | cg00504705 | -2.383687108 | PCDHA1 | Body | Island |
|  |  | cg02004851 | 12.07402308 | PCDHA1 | Body | N_Shore |
|  |  | cg09852127 | -6.754210675 | PCDHA1 | Body | N_Shore |
|  |  | cg11268983 | -0.269047156 | PCDHA1 | Body | N_Shore |
|  |  | cg14557699 | -0.174656362 | PCDHA1 | Body | N_Shore |
|  |  | cg19596110 | -0.015585352 | PCDHA1 | Body | Island |
| PCDHA10 | chr5:140,855,897-141,012,347 | cg00275741 | 0.680151182 | PCDHA10 | Body | N_Shore |
|  |  | cg09852127 | -6.754210675 | PCDHA10 | Body | N_Shore |
|  |  | cg11268983 | -0.269047156 | PCDHA10 | Body | N_Shore |
|  |  | cg14557699 | -0.174656362 | PCDHA10 | Body | N_Shore |
| PCDHA11 | chr5:140,868,957-141,012,347 | cg00275741 | 0.680151182 | PCDHA11 | Body | N_Shore |
|  |  | cg09852127 | -6.754210675 | PCDHA11 | Body | N_Shore |
|  |  | cg11268983 | -0.269047156 | PCDHA11 | Body | N_Shore |
|  |  | cg14557699 | -0.174656362 | PCDHA11 | Body | N_Shore |
| PCDHA12 | chr5:140,875,308-141,012,347 | cg00275741 | 0.680151182 | PCDHA12 | Body | N_Shore |
| **GeneSymbol_inner** | **Gene_pos** | **CpG** | **Score** | **GeneSymbol** | **Group** | **RelationToIsland** |
| PCDHA12 | chr5:140,875,308-141,012,347 | cg09852127 | -6.754210675 | PCDHA12 | TSS200 | N_Shore |
|  |  | cg11268983 | -0.269047156 | PCDHA12 | Body | N_Shore |
|  |  | cg14557699 | -0.174656362 | PCDHA12 | TSS200 | N_Shore |
| PCDHA13 | chr5:140,882,124-141,012,347 | cg00275741 | 0.680151182 | PCDHA13 | TSS200 | N_Shore |
|  |  | cg11268983 | -0.269047156 | PCDHA13 | 1stExon | N_Shore |
| PCDHA2 | chr5:140,794,852-141,012,347 | cg00275741 | 0.680151182 | PCDHA2 | Body | N_Shore |
|  |  | cg00504705 | -2.383687108 | PCDHA2 | Body | Island |
|  |  | cg02004851 | 12.07402308 | PCDHA2 | Body | N_Shore |
|  |  | cg09852127 | -6.754210675 | PCDHA2 | Body | N_Shore |
|  |  | cg11268983 | -0.269047156 | PCDHA2 | Body | N_Shore |
|  |  | cg14557699 | -0.174656362 | PCDHA2 | Body | N_Shore |
|  |  | cg19596110 | -0.015585352 | PCDHA2 | Body | Island |
| PCDHA3 | chr5:140,801,057-141,012,347 | cg00275741 | 0.680151182 | PCDHA3 | Body | N_Shore |
|  |  | cg00504705 | -2.383687108 | PCDHA3 | Body | Island |
|  |  | cg02004851 | 12.07402308 | PCDHA3 | Body | N_Shore |
|  |  | cg09852127 | -6.754210675 | PCDHA3 | Body | N_Shore |
|  |  | cg11268983 | -0.269047156 | PCDHA3 | Body | N_Shore |
|  |  | cg14557699 | -0.174656362 | PCDHA3 | Body | N_Shore |
|  |  | cg19596110 | -0.015585352 | PCDHA3 | Body | Island |
| PCDHA4 | chr5:140,807,068-141,012,347 | cg00275741 | 0.680151182 | PCDHA4 | Body | N_Shore |
|  |  | cg00504705 | -2.383687108 | PCDHA4 | Body | Island |
|  |  | cg02004851 | 12.07402308 | PCDHA4 | Body | N_Shore |
| PCDHA4 | chr5:140,807,068-141,012,347 | cg09852127 | -6.754210675 | PCDHA4 | Body | N_Shore |
|  |  | cg11268983 | -0.269047156 | PCDHA4 | Body | N_Shore |
|  |  | cg14557699 | -0.174656362 | PCDHA4 | Body | N_Shore |
| **GeneSymbol_inner** | **Gene_pos** | **CpG** | **Score** | **GeneSymbol** | **Group** | **RelationToIsland** |
| PCDHA4 | chr5:140,807,068-141,012,347 | cg19596110 | -0.015585352 | PCDHA4 | Body | Island |
| PCDHA5 | chr5:140,821,613-141,012,347 | cg00275741 | 0.680151182 | PCDHA5 | Body | N_Shore |
|  |  | cg00504705 | -2.383687108 | PCDHA5 | Body | Island |
|  |  | cg02004851 | 12.07402308 | PCDHA5 | Body | N_Shore |
|  |  | cg09852127 | -6.754210675 | PCDHA5 | Body | N_Shore |
|  |  | cg11268983 | -0.269047156 | PCDHA5 | Body | N_Shore |
|  |  | cg14557699 | -0.174656362 | PCDHA5 | Body | N_Shore |
|  |  | cg19596110 | -0.015585352 | PCDHA5 | Body | Island |
| PCDHA6 | chr5:140,827,960-141,012,347 | cg00275741 | 0.680151182 | PCDHA6 | Body | N_Shore |
|  |  | cg00504705 | -2.383687108 | PCDHA6 | Body | Island |
|  |  | cg02004851 | 12.07402308 | PCDHA6 | TSS200 | N_Shore |
|  |  | cg09852127 | -6.754210675 | PCDHA6 | Body | N_Shore |
|  |  | cg11268983 | -0.269047156 | PCDHA6 | Body | N_Shore |
|  |  | cg14557699 | -0.174656362 | PCDHA6 | Body | N_Shore |
|  |  | cg19596110 | -0.015585352 | PCDHA6 | Body | Island |
| PCDHA7 | chr5:140,834,269-141,012,347 | cg00275741 | 0.680151182 | PCDHA7 | Body | N_Shore |
|  |  | cg00504705 | -2.383687108 | PCDHA7 | Body | Island |
|  |  | cg09852127 | -6.754210675 | PCDHA7 | Body | N_Shore |
|  |  | cg11268983 | -0.269047156 | PCDHA7 | Body | N_Shore |
|  |  | cg14557699 | -0.174656362 | PCDHA7 | Body | N_Shore |
|  |  | cg19596110 | -0.015585352 | PCDHA7 | Body | Island |
| PCDHA8 | chr5:140,841,187-141,012,347 | cg00275741 | 0.680151182 | PCDHA8 | Body | N_Shore |
| PCDHA8 | chr5:140,841,187-141,012,347 | cg00504705 | -2.383687108 | PCDHA8 | 1stExon | Island |
| PCDHA8 | chr5:140,841,187-141,012,347 | cg09852127 | -6.754210675 | PCDHA8 | Body | N_Shore |
|  |  | cg11268983 | -0.269047156 | PCDHA8 | Body | N_Shore |
| **GeneSymbol_inner** | **Gene_pos** | **CpG** | **Score** | **GeneSymbol** | **Group** | **RelationToIsland** |
| PCDHA8 | chr5:140,841,187-141,012,347 | cg14557699 | -0.174656362 | PCDHA8 | Body | N_Shore |
|  |  | cg19596110 | -0.015585352 | PCDHA8 | 1stExon | Island |
| PCDHA9 | chr5:140,848,382-141,012,347 | cg00275741 | 0.680151182 | PCDHA9 | Body | N_Shore |
|  |  | cg09852127 | -6.754210675 | PCDHA9 | Body | N_Shore |
|  |  | cg11268983 | -0.269047156 | PCDHA9 | Body | N_Shore |
|  |  | cg14557699 | -0.174656362 | PCDHA9 | Body | N_Shore |
| PLCB4 | chr20:9,069,087-9,480,808 | cg18870258 | 115.1982988 | PLCB4 | 3'UTR | OpenSea |
| PRICKLE2 | chr3:64,092,236-64,225,466 | cg18450254 | 49.69152436 | PRICKLE2 | 5'UTR | OpenSea |
| PRTG | chr15:55,611,544-55,743,152 | cg03450593 | 94.07358063 | PRTG | Body | OpenSea |
| PSD3 | chr8:18,527,303-19,013,703 | cg22276811 | 75.56396882 | PSD3 | Body | OpenSea |
| RAMP1 | chr2:237,859,623-237,912,106 | cg18361425 | 107.923944 | RAMP1 | Body | OpenSea |
| RBFOX3 | chr17:79,089,345-79,611,051 | cg15757793 | 99.10918587 | RBFOX3 | 5'UTR | OpenSea |
| RBP1 | chr3:139,517,438-139,539,742 | cg15288618 | 77.18299419 | RBP1 | Body | Island |
|  |  | cg23448348 | 70.22616432 | RBP1 | Body | Island |
| RPTOR | chr17:80,544,838-80,966,368 | cg16438182 | 143.4157906 | RPTOR | Body | S_Shore |
| SATB2-AS1 | chr2:199,457,837-199,476,504 | cg15981734 | 76.1921609 | SATB2-AS1 | Body | N_Shore |
| SIX3 | chr2:44,941,702-44,946,071 | cg14673618 | 51.55821451 | SIX3 | Body | Island |
| SLC20A2 | chr8:42,416,475-42,501,231 | cg22855020 | 82.21785274 | SLC20A2 | 5'UTR | OpenSea |
| SLC2A2 | chr3:170,996,347-171,026,720 | cg24302235 | 90.39773342 | SLC2A2 | TSS1500 | Island |
| SLPI | chr20:45,252,239-45,254,564 | cg08548498 | 89.19595122 | SLPI | TSS1500 | OpenSea |
| SOX2-OT | chr3:180,989,770-181,791,029 | cg21453451 | 64.48987539 | SOX2-OT | Body | S_Shore |
| SPATA2 | chr20:49,903,391-49,915,529 | cg19277884 | 51.6298945 | SPATA2 | 5'UTR;TSS200 | N_Shore |
| TCF23 | chr2:27,149,004-27,156,974 | cg15326452 | 122.8713704 | TCF23 | TSS200 | N_Shore |
| THBD | chr20:23,045,633-23,049,672 | cg16339238 | 67.15607699 | THBD | 1stExon | Island |
| TMEM106A | chr17:43,211,875-43,220,041 | cg18222083 | 52.50244246 | TMEM106A | 5'UTR | Island |
| GeneSymbol_inner | Gene_pos | CpG | Score | GeneSymbol | Group | RelationToIsland |
| TOX2 | chr20:43,914,852-44,069,616 | cg03011535 | 54.51092498 | TOX2 | 5'UTR;1stExon;Body | Island |
| TRANK1 | chr3:36,826,819-36,945,054 | cg09550909 | 54.53398199 | TRANK1 | Body | OpenSea |
| WFDC2 | chr20:45,469,753-45,481,532 | cg06385187 | 56.35032629 | WFDC2 | TSS200 | Island |
| WNK4 | chr17:42,780,610-42,797,066 | cg14457782 | 64.91922395 | WNK4 | Body | Island |
| ZIC1 | chr3:147,409,365-147,416,719 | cg18297437 | 57.89609371 | ZIC1 | Body | Island |
| ZIC4 | chr3:147,386,046-147,406,543 | cg16790847 | 50.22814238 | ZIC4 | 5'UTR;TSS1500;TSS200 | N_Shelf |
|  |  | cg17569743 | 60.41974136 | ZIC4 | Body | Island |
|  |  | cg02025737 | 226.8421349 |  |  | OpenSea |
|  |  | cg02639540 | 57.37775534 |  |  | S_Shore |
|  |  | cg03147324 | 106.878972 |  |  | OpenSea |
|  |  | cg03991106 | 60.53000913 |  |  | OpenSea |
|  |  | cg06060522 | 49.68171945 |  |  | Island |
|  |  | cg07864976 | 61.21393801 |  |  | OpenSea |
|  |  | cg10244340 | 49.55638612 |  |  | Island |
|  |  | cg12198140 | 97.60587768 |  |  | OpenSea |
|  |  | cg12974388 | 57.35766582 |  |  | Island |
|  |  | cg14262681 | 91.25989403 |  |  | N_Shore |
|  |  | cg15054725 | 66.45759483 |  |  | Island |
|  |  | cg15063366 | 143.6605142 |  |  | OpenSea |
|  |  | cg15504461 | 79.5633789 |  |  | N_Shore |
|  |  | cg15745944 | 67.63296078 |  |  | OpenSea |
|  |  | cg16175703 | 60.34206527 |  |  | OpenSea |
|  |  | cg16683770 | 111.711225 |  |  | Island |
|  |  | cg17504394 | 76.61640809 |  |  | OpenSea |
|  |  | cg18244601 | 109.0362535 |  |  | Island |
| **GeneSymbol_inner** | **Gene_pos** | **CpG** | **Score** | **GeneSymbol** | **Group** | **RelationToIsland** |
|  |  | cg18759732 | 51.16691599 |  |  | OpenSea |
|  |  | cg19112357 | 96.27997929 |  |  | OpenSea |
|  |  | cg19112895 | 70.36615662 |  |  | N_Shelf |
|  |  | cg19623418 | 126.6907648 |  |  | N_Shore |
|  |  | cg19709468 | 107.6405217 |  |  | N_Shore |
|  |  | cg20761810 | 55.67127829 |  |  | OpenSea |
|  |  | cg21398280 | 50.72303931 |  |  | OpenSea |
|  |  | cg22369879 | 72.09651059 |  |  | OpenSea |
|  |  | cg22973468 | 132.1359674 |  |  | Island |
|  |  | cg23322933 | 65.16038255 |  |  | Island |
|  |  | cg23324048 | 49.92253517 |  |  | Island |
|  |  | cg02326806 | 72.38656808 |  |  | Island |
|  |  | cg09775533 | 72.45696758 |  |  | Island |
|  |  | cg15316843 | 61.33222705 |  |  | Island |
|  |  | cg19271111 | 61.26995826 |  |  | S_Shore |
|  |  | cg22457769 | 73.01110009 |  |  | S_Shore |
|  |  | cg00655552 | 48.15454968 |  |  | OpenSea |
|  |  | cg03702533 | 48.27294677 |  |  | OpenSea |

**Supplementary Table 9.** Enrichment analysis of genes mapped by the top 100 probes for breast cancer.

| **GO Term** | **Term_id** | **Adjusted_P_Value** | **Negative_log10** | **Term_size** |
| --- | --- | --- | --- | --- |
| homophilic cell adhesion via plasma membrane adhesion molecules | GO:0007156 | 5.0E-14 | 13.30 | 168 |
| cell-cell adhesion via plasma-membrane adhesion molecules | GO:0098742 | 8.7E-14 | 13.06 | 280 |
| cell-cell adhesion | GO:0098609 | 1.0E-09 | 8.98 | 946 |
| calcium ion binding | GO:0005509 | 1.0E-09 | 8.98 | 726 |
| multicellular organism development | GO:0007275 | 2.6E-07 | 6.58 | 4643 |
| anatomical structure development | GO:0048856 | 5.3E-07 | 6.28 | 5899 |
| cell adhesion | GO:0007155 | 6.5E-07 | 6.19 | 1512 |
| multicellular organismal process | GO:0032501 | 1.1E-06 | 5.97 | 7669 |
| nervous system development | GO:0007399 | 1.6E-06 | 5.79 | 2531 |
| developmental process | GO:0032502 | 2.0E-06 | 5.71 | 6453 |

**Supplementary Table 10.** Enrichment analysis of genes mapped by the top 100 probes for prostate cancer.

| **GO Term** | **Term_id** | **Adjusted_P_Value** | **Negative_log10** | **Term_size** |
| --- | --- | --- | --- | --- |
| homophilic cell adhesion via plasma membrane adhesion molecules | GO:0007156 | 8.84E-16 | 15.05 | 168 |
| cell-cell adhesion via plasma-membrane adhesion molecules | GO:0098742 | 1.97E-12 | 11.70 | 280 |
| calcium ion binding | GO:0005509 | 7.90E-10 | 9.10 | 726 |
| cell adhesion | GO:0007155 | 5.97E-08 | 7.22 | 1511 |
| nervous system development | GO:0007399 | 1.91E-07 | 6.72 | 2541 |
| multicellular organismal process | GO:0032501 | 2.80E-07 | 6.55 | 7234 |
| multicellular organism development | GO:0007275 | 8.57E-07 | 6.07 | 4658 |
| cell-cell adhesion | GO:0098609 | 8.61E-07 | 6.07 | 948 |
| anatomical structure development | GO:0048856 | 7.98E-06 | 5.10 | 5924 |
| developmental process | GO:0032502 | 2.62E-05 | 4.58 | 6478 |

**Supplementary Table 11.** Standardized expression values of top 100 probes mapped to 68 genes across major cell types in breast cancer.

| **Gene Symbol** | **B-cells** | **CAFs** | **Cancer Epithelial** | **Endothelial** | **Myeloid** | **Normal Epithelial** | **PVL** | **Plasmablasts** | **T-cells** |
| --- | --- | --- | --- | --- | --- | --- | --- | --- | --- |
| RPTOR | 0.650012 | 0.437765 | 1 | 0.643569 | 0.596607 | 0.804029 | 0.207083 | 0 | 0.60336 |
| PLCB4 | 0.008216 | 0.201355 | 0.262725 | 1 | 0.013565 | 0.13264 | 0.194008 | 0.001335 | 0 |
| MYOM2 | 0.194187 | 1 | 0.041398 | 0.362172 | 0.23969 | 0.391032 | 0.442063 | 0 | 0.691588 |
| PRTG | 0.015765 | 1 | 0.219284 | 0.866436 | 0 | 0.656007 | 0.323972 | 0.001134 | 0.005723 |
| CPQ | 0.028683 | 1 | 0.107939 | 0.343552 | 0.296185 | 0.315648 | 0.584613 | 0 | 0.075027 |
| PCDH10 | 0.025335 | 0.184242 | 1 | 0.272221 | 0.057173 | 0.342418 | 0.720657 | 0 | 0.001173 |
| NCKAP5 | 0.00491 | 0.049082 | 0.257785 | 0.059584 | 0.082633 | 0.317127 | 1 | 0.001625 | 0 |
| TRANK1 | 1 | 0.664932 | 0 | 0.398224 | 0.384528 | 0.064372 | 0.257343 | 0.246172 | 0.809733 |
| NRXN1 | 0 | 0.008458 | 0.029392 | 0.02361 | 0.001094 | 1 | 0 | 0 | 0.001743 |
| PRICKLE2 | 0.001681 | 1 | 0.175888 | 0.446761 | 0.009984 | 0.378426 | 0.55286 | 0.007476 | 0 |
| CSMD1 | 0 | 0.21749 | 0.715608 | 0.812961 | 0.052084 | 1 | 0.145169 | 0.063953 | 0.02187 |
| DNAH1 | 0.392915 | 0.223133 | 0.001173 | 1 | 0.874053 | 0.238128 | 0 | 0.019139 | 0.603219 |
| PCDHA3 | 0 | 1 | 0.058266 | 0.259929 | 0 | 0.548332 | 0.80121 | 0 | 0 |
| PCDHA4 | 0 | 0.359769 | 1 | 0.228802 | 0.020425 | 0.191391 | 0.172917 | 0 | 0.041275 |
| PCDHA6 | 0 | 0.370637 | 0.415474 | 0.108835 | 0 | 1 | 0.400084 | 0 | 0 |
| DNAH17 | 0.077301 | 0 | 0.087241 | 0.045607 | 1 | 0.009338 | 0.039884 | 0.006699 | 0.023045 |
| PCDHA9 | 0 | 0.846096 | 0.72789 | 0.269593 | 0.01432 | 0.486496 | 1 | 0 | 0.142281 |
| PCDHA10 | 0 | 0.674048 | 0.755783 | 0.095101 | 0.026425 | 1 | 0.710529 | 0.010504 | 0.010225 |
| PCDHA11 | 0 | 1 | 0.849592 | 0 | 0 | 0.486424 | 0.443395 | 0 | 0 |

**Supplementary Table 12.** Summary of the top 20 genes ranked by model explainable ability, including somatic mutation rates, normalized expression levels in cancer single cells, and significance values from survival and differential expression analyses.

| **Rank** | **Gene** | **Somatic Mutation Rate** | **Normalized Expression Value in Cancer** | **Survival Analysis P-value** | **DEG P-value** |
| --- | --- | --- | --- | --- | --- |
| 1 | RPTOR | 4.00% | 1 | 8.60E-03 | 2.55E-02 |
| 2 | PLCB4 | 2.90% | 0.2627 | 1.86E-01 | < 2.2E-16 |
| 3 | MYOM2 | 6.00% | 0.0414 | 2.07E-01 | 9.10E-08 |
| 4 | PRTG | 1.50% | 0.2193 | 3.00E-04 | < 2.2E-16 |
| 5 | CPQ | 13.00% | 0.1079 | 8.40E-05 | < 2.2E-16 |
| 6 | PCDH10 | 1.30% | 1 | 7.33E-02 | 3.12E-04 |
| 7 | NCKAP5 | 1.60% | 0.2578 | 1.90E-05 | 2.87E-01 |
| 8 | TRANK1 | 1.40% | 0 | 1.40E-13 | < 2.2E-16 |
| 9 | NRXN1 | 2.00% | 0.0294 | 1.60E-02 | 9.03E-13 |
| 10 | PRICKLE2 | 1.00% | 0.1759 | 2.00E-04 | < 2.2E-16 |
| 11 | CSMD1 | 10.00% | 0.7156 | 4.21E-01 | < 2.2E-16 |
| 12 | DNAH1 | 2.00% | 0.0012 | < 1E-16 | < 2.2E-16 |
| 13 | PCDHA3 | 1.20% | 0.0583 | 1.23E-01 | < 2.2E-16 |
| 14 | PCDHA4 | 1.00% | 1 | 4.29E-01 | 7.64E-07 |
| 15 | PCDHA6 | 1.20% | 0.4155 | 4.00E-04 | 4.21E-03 |
| 16 | DNAH17 | 8.00% | 0.0872 | 8.67E-01 | 1.16E-07 |
| 17 | PCDHA9 | 1.30% | 0.7279 | 4.30E-05 | 2.73E-04 |
| 18 | PCDHA10 | 1.30% | 0.7558 | 3.83E-02 | < 2.2E-16 |
| 19 | PCDHA11 | 1.40% | 0.8496 | 4.29E-01 | 1.14E-02 |
| 20 | PCDHA13 | 1.10% | 0.5457 | 4.29E-01 | 1.16E-02 |


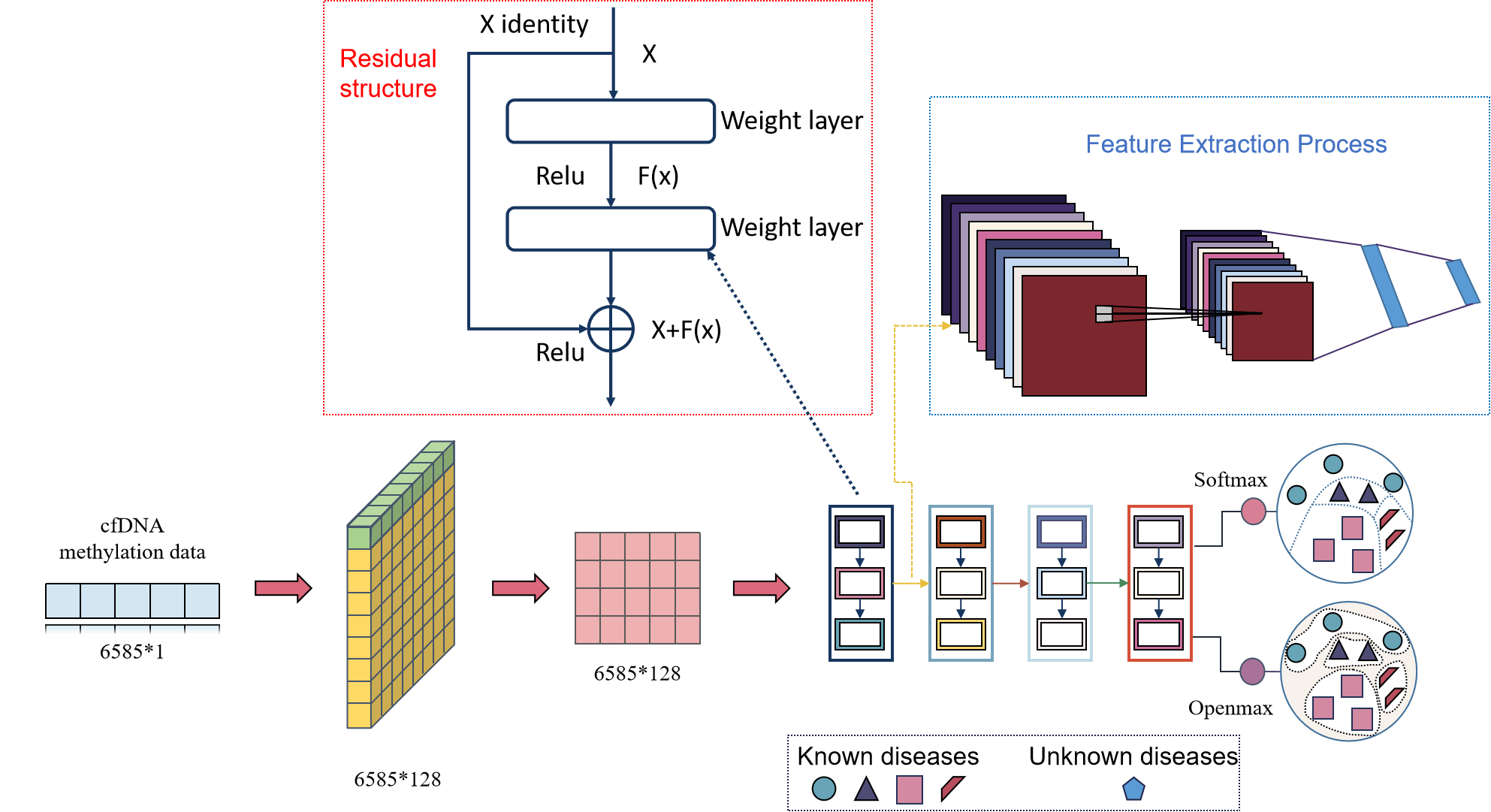


**Supplementary Figure 1.** Framework of the full ResNet architecture with integrated PPE module.


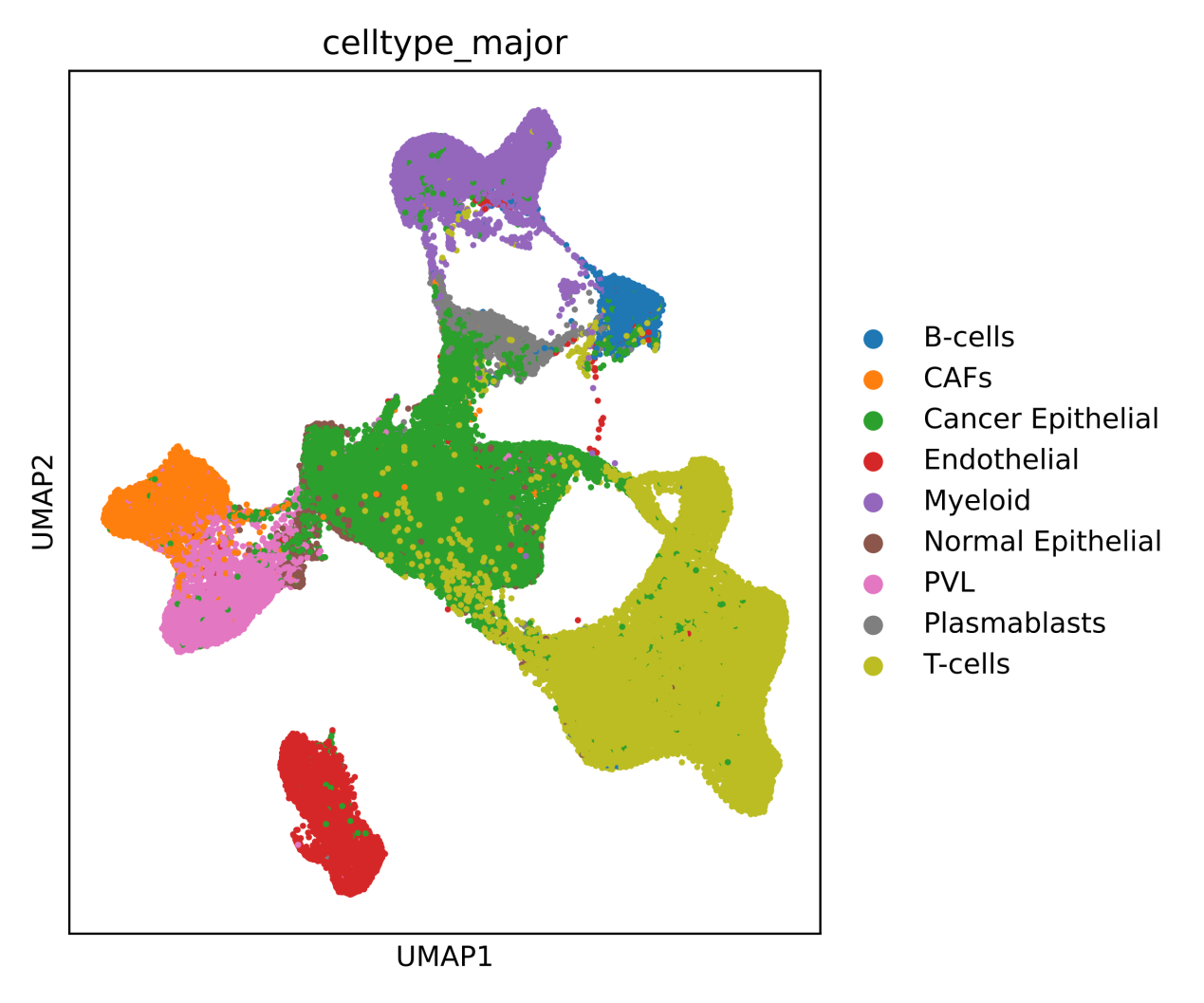


**Supplementary Figure 2.** Uniform manifold approximation and projection map (UMAP) of 100064 cells color-coded for the indicated cell type.

**Abreviations:** CAFs, cancer-associated fibroblasts; PVL, perivascular lymphocytes.


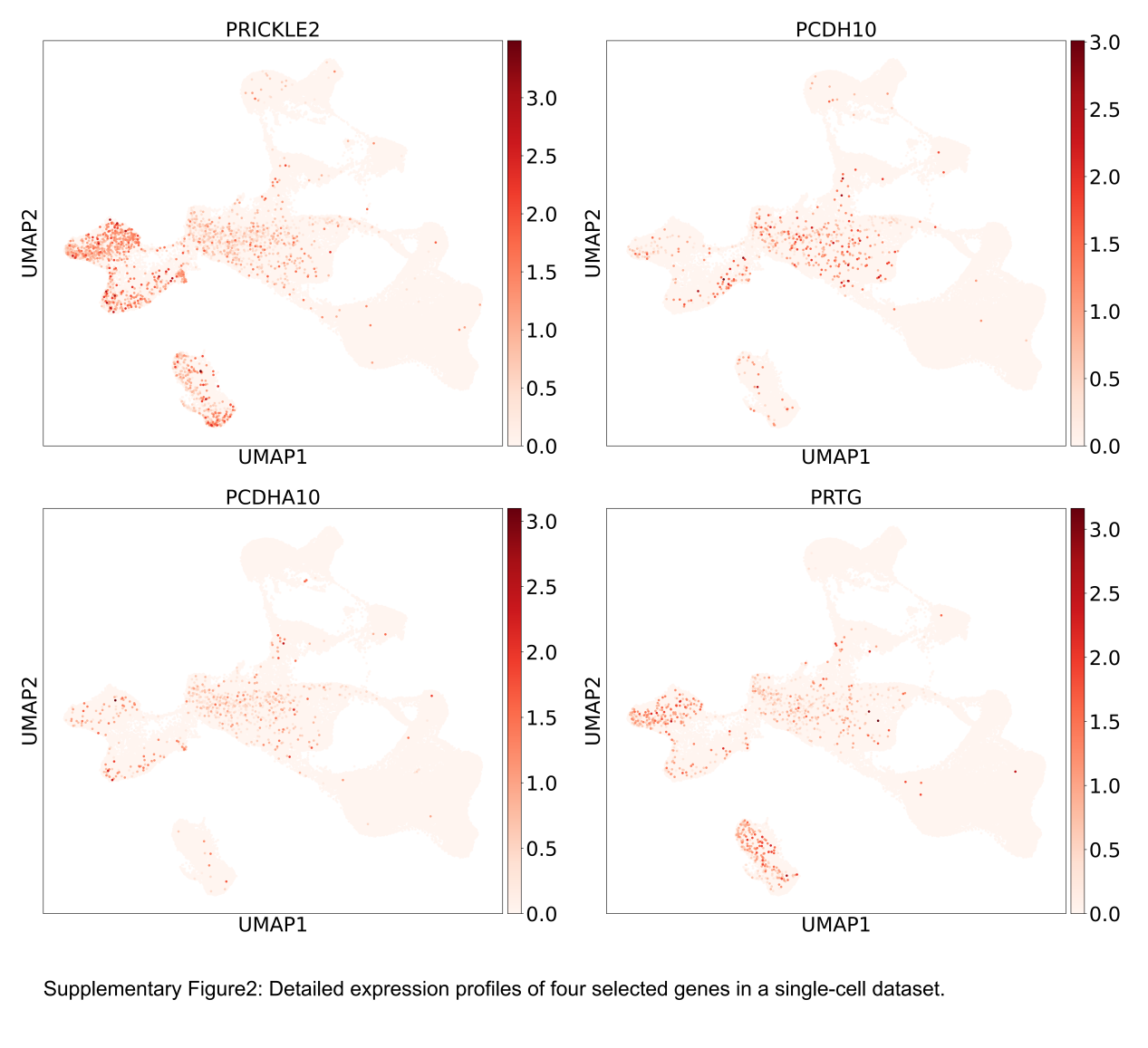


**Supplementary Figure 3.** Expression of *PRICKLE2*, *PCDH10*, *PCDHA10*, and *PRTG* genes in single-cell dataset.


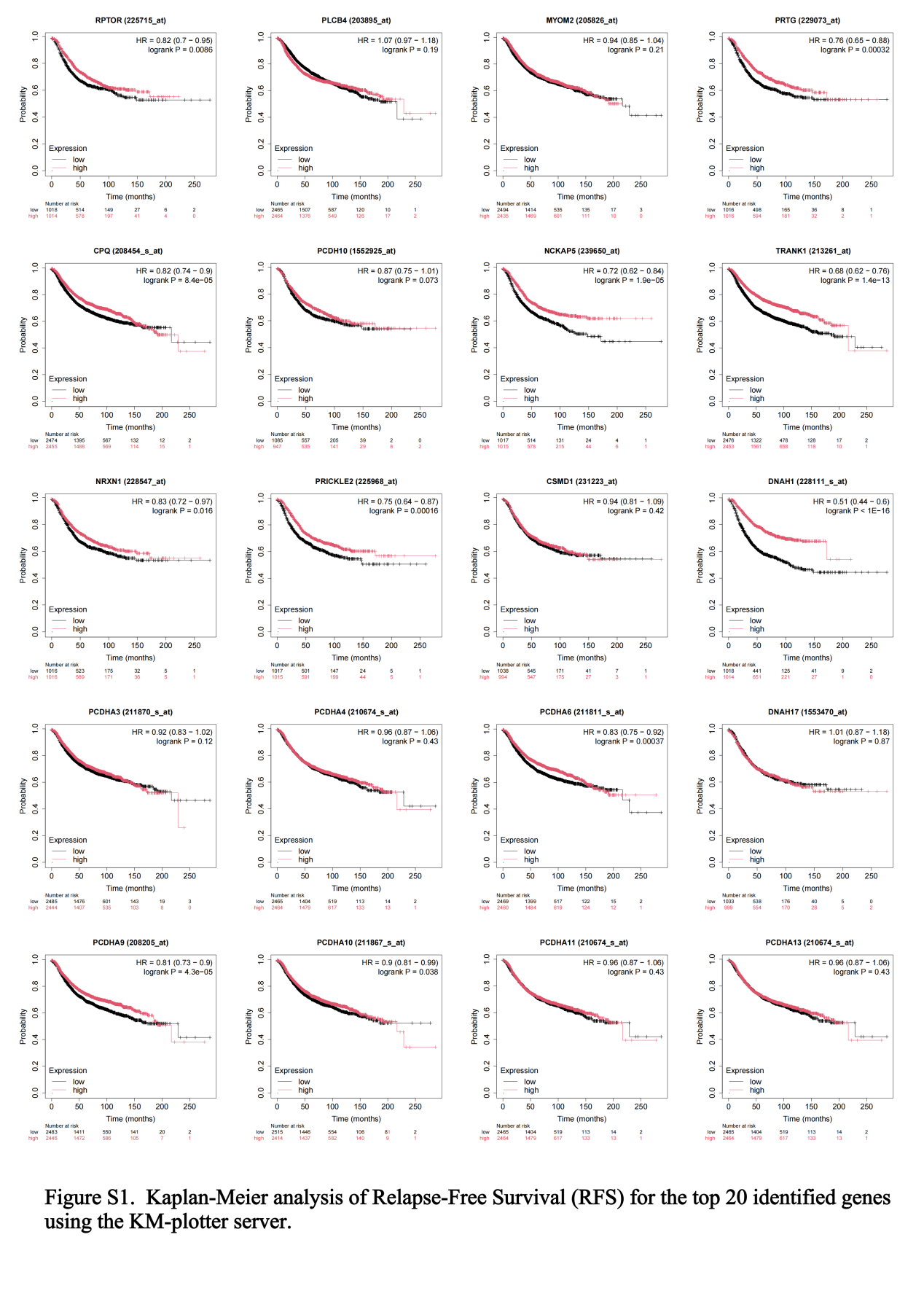


**Supplementary Figure 4.** Kaplan–Meier analysis of relapse-free survival (RFS) for the top 20 genes with the highest mutation rates among the top 100 probes mapped to high-impact genes.
